# Acute myeloid leukemia maturation lineage influences residual disease and relapse following differentiation therapy

**DOI:** 10.1038/s41467-021-26849-w

**Published:** 2021-11-11

**Authors:** Steven Ngo, Ethan P. Oxley, Margherita Ghisi, Maximilian M. Garwood, Mark D. McKenzie, Helen L. Mitchell, Peter Kanellakis, Olivia Susanto, Michael J. Hickey, Andrew C. Perkins, Benjamin T. Kile, Ross A. Dickins

**Affiliations:** 1 Australian Centre for Blood Diseases, Monash University, 99 Commercial Rd, Melbourne, VIC 3004 Australia; 2 Walter and Eliza Hall Institute of Medical Research, 1G Royal Parade, Parkville, VIC 3052 Australia; 3 Baker Heart and Diabetes Institute, 75 Commercial Rd, Melbourne, VIC 3004 Australia; 4 Centre for Inflammatory Diseases, Monash University Department of Medicine, Monash Medical Centre, 246 Clayton Rd, Clayton, VIC 3168 Australia; 5 Anatomy and Developmental Biology, Monash Biomedicine Discovery Institute, Monash University, Clayton, VIC 3800 Australia

**Keywords:** Acute myeloid leukaemia, Cancer therapeutic resistance

## Abstract

Acute myeloid leukemia (AML) is a malignancy of immature progenitor cells. AML differentiation therapies trigger leukemia maturation and can induce remission, but relapse is prevalent and its cellular origin is unclear. Here we describe high resolution analysis of differentiation therapy response and relapse in a mouse AML model. Triggering leukemia differentiation in this model invariably produces two phenotypically distinct mature myeloid lineages in vivo. Leukemia-derived neutrophils dominate the initial wave of leukemia differentiation but clear rapidly and do not contribute to residual disease. In contrast, a therapy-induced population of mature AML-derived eosinophil-like cells persists during remission, often in extramedullary organs. Using genetic approaches we show that restricting therapy-induced leukemia maturation to the short-lived neutrophil lineage markedly reduces relapse rates and can yield cure. These results indicate that relapse can originate from therapy-resistant mature AML cells, and suggest differentiation therapy combined with targeted eradication of mature leukemia-derived lineages may improve disease outcome.

## Abstract (web summary)

Differentiation therapy induces the maturation and clearance of acute myeloid leukemia cells. Here, using a mouse model, the authors show that a specific lineage of mature leukemia-derived cells persists during remission and is responsible for disease relapse.

## Introduction

AML is the malignant transformation of myeloid progenitor cells arrested in an immature and intrinsically proliferative state. Most AML patients are treated with cytotoxic chemotherapy to induce immature leukemia cell death, but this has dose-limiting side effects and most patients ultimately relapse with resistant disease. In contrast, differentiation therapy triggers AML maturation and subsequent turnover of mature leukemia-derived cells, engaging mechanisms that normally produce and clear over 100 billion mature myeloid cells (predominantly neutrophils) daily^1^. Differentiation therapy has proven particularly effective in acute promyelocytic leukemia (APL), an AML subtype driven by the PML-RARA fusion oncoprotein^2^. All-trans retinoic acid (ATRA) therapy triggers PML-RARA degradation and *en masse* leukemia maturation, resulting in acute AML-derived leukocytosis within 1–3 weeks and an associated differentiation syndrome in ~25% of patients^3,4^. Historically most patients achieved remission on ATRA-based therapy but up to 25% relapsed with immature disease^5^. Combining ATRA with arsenic trioxide has improved response rates and overall five years of APL survival now exceeds 90%^2,6^. ATRA and other RARA agonists can also induce differentiation of some non-APL AML subtypes^2,7,8^.

In the last decade several additional agents that induce maturation across AML subtypes have entered (pre-) clinical development^9^. These include targeted inhibitors of FLT3^10,11^, HDACs^12^, DHODH^13,14^, and LSD1^15,16^. Foremost among emerging differentiation therapies are ivosidenib (AG-120) and enasidenib (AG-221), clinically approved oral inhibitors of mutant isocitrate dehydrogenase 1 (IDH1) and IDH2 respectively. Encoded by somatic mutations found in ~20% of AML patients, these defective enzymes produce the oncometabolite 2-hydroxyglutarate (2HG) that contributes to a myeloid differentiation block^17,18^. Clinical response to mutant IDH1/2 inhibitors is associated with AML maturation that yields functional IDH-mutant neutrophils^19–22^, with differentiation syndrome in ~10% of these patients^22,23^. However almost all patients treated with mutant IDH1/2 inhibitors eventually relapse, even after complete remission^20,22^.

Genomic studies of APL samples from patients treated between ~1985 to ~1995 with ATRA alone or ATRA/chemotherapy have identified two distinct modes of relapse following differentiation therapy^24,25^. In most cases relapse resembles diagnostic disease but with additional mutations that impede treatment response^24,26^. Indeed, up to one third of APL relapses harbor mutations in the ATRA binding domain of the PML-RARA oncoprotein that reduce ATRA-induced degradation^27–29^. A second minor group of post-ATRA relapses derive from ancestral PML-RARA expressing clones^24^. More recent studies show that clinical resistance to enasidenib and ivosidenib also arises through multiple mechanisms, and relapse is often associated with acquired mutations (e.g., in *RUNX1*) that reimpose a myeloid differentiation block^30^. In a minority of patients, relapse on treatment is accompanied by rising 2HG levels driven by drug-resistant second-site mutations in IDH1 or IDH2^31^ or ‘isoform switching’ mutations in the untargeted IDH isoform^30,32^.

Although genetic aberrations associated with AML differentiation therapy resistance are being rapidly uncovered, the cellular origin of relapse remains poorly understood. In principle, if leukemia maturation is unidirectional and irreversible then relapse must arise from selection of immature leukemia cells with pre-existing mutations that prevent therapy-induced maturation. However recent analysis of a mouse AML model driven by reversible RNAi-mediated knockdown of the myeloid transcription factor PU.1 demonstrated interconversion between an immature leukemogenic state and a mature non-leukemogenic state based on PU.1 suppression or restoration respectively^33,34^. Similarly, ATRA-induced APL maturation can also be reversed upon treatment cessation^33^. This unforeseen maturational plasticity raises the possibility that mature AML cells may contribute to disease relapse following differentiation therapy. Examining this in AML patients is challenging due to phenotypic similarities between mature leukemia-derived cells and their normal myeloid counterparts. Hence in this study we have generated a tractable mouse AML model permitting high resolution analysis of differentiation therapy response, remission, and relapse.

## Results

### Tracking AML differentiation therapy response and relapse in vivo

The mouse AML246 leukemia model is driven by knockdown of PU.1, a myeloid transcription factor directly inhibited by several recurrent AML driver proteins including PML-RARA, AML1-ETO, and NPM1c. In AML246, which was generated on a *Trp53*^*−/−*^ background and has an acquired activating Kit mutation, a tetracycline-regulated promoter reversibly controlled by the tTA transactivator drives PU.1 short hairpin RNA (shRNA) expression linked to green fluorescent protein (GFP)^33^. Untreated AML246 leukemia cells are therefore PU.1-low, GFP^HIGH^, immature, proliferative, and leukemogenic, whereas doxycycline (Dox) treatment restores endogenous PU.1 and triggers in vivo leukemia differentiation and sustained clearance^33^. Despite leukemia maturation, after several months on Dox mice invariably relapse with immature leukemia resembling the original disease^33^. This genetic model therefore provides a unique opportunity to investigate the origins of relapse following AML differentiation therapy.

**Fig. 1:**
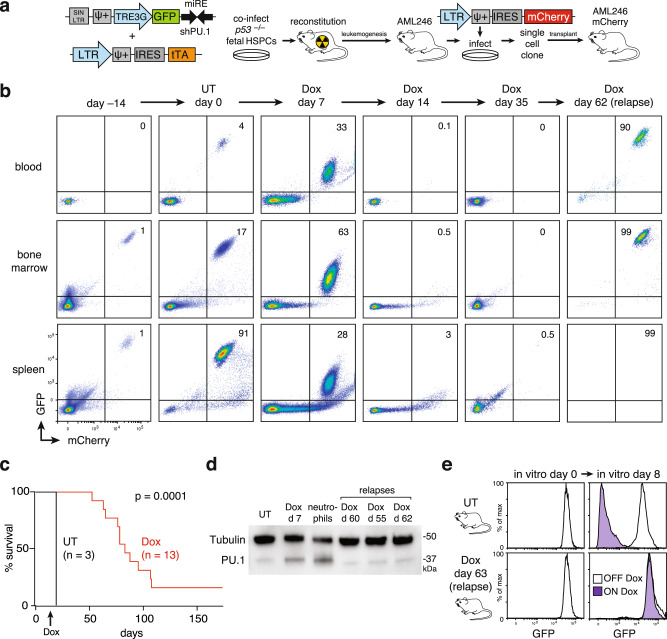
An in vivo model of AML differentiation therapy regression, remission, and relapse. **a** Strategy for generating AML246-Cherry. **b** Flow cytometry analysis of disease burden (mCherry^+^ cells) prior to and following Dox treatment in vivo. Data are from a representative mouse at each time point. Axis scales are shown on the lower left plot only. UT, untreated. All mice used in the study are listed in Supplementary Table 1. **c** Kaplan–Meier survival analysis of mice transplanted with AML246-Cherry cells at day 0 and either left untreated (UT; *n* = 3) or Dox treated (*n* = 13) upon leukemia establishment as indicated. *p* = 0.0001, Mantel-Cox test. Source data are provided as a Source Data file. **d** PU.1 immunoblotting of AML246-Cherry cells from untreated mice, 8 day Dox treated mice, or mice relapsing on Dox, compared to control normal mouse Cd11b^+^Gr1^+^ neutrophils. Tubulin loading control. The blot was performed once. Source data are provided as a Source Data file. **e** GFP flow cytometry of GFP^+^mCherry^+^ AML246-Cherry cells isolated from the bone marrow of mice that had relapsed during Dox treatment. Cells were cultured with or without Dox for 8 days then analyzed. AML246-Cherry cells isolated from an untreated mouse (GFP^+^mCherry^+^ but Dox-sensitive) were used as controls.

Since Dox treatment of mice harboring AML246 induces differentiation but also shuts off GFP, maturing leukemia cells are often indistinguishable from normal host myeloid lineage cells. To allow high resolution leukemia tracking in vivo we transduced AML246 cells with a retroviral vector stably expressing mCherry. An mCherry^+^ clone derived from a single AML246 cell (designated AML246-Cherry) was expanded in culture then transplanted into immunocompromised *Rag1*^*−/−*^ mice to permit engraftment and in vivo expansion (Fig. 1a). AML246-Cherry recipient mice developed aggressive mCherry^+^GFP^HIGH^ leukemia within 3–4 weeks (Fig. 1b).

To trigger AML246-Cherry differentiation we initiated Dox treatment when mice had a peripheral blood mCherry^+^ leukemia burden of ~2–10%. As expected, Dox feed suppressed GFP and restored PU.1 protein expression in leukemia cells but mCherry fluorescence remained high (Fig. 1b). After 1 week on Dox the proportion of leukemia cells in the blood, bone marrow, and spleen continued to rise in line with rapid disease expansion pre-Dox, however most leukemia cells ceased proliferating consistent with differentiation (Fig. 1b and Supplementary Fig. 1a, b). After 2 weeks of Dox treatment, mCherry^+^GFP^LOW^ leukemia-derived cells were undetectable in blood and had plummetted in bone marrow and spleen indicating widespread disease clearance (Fig. 1b). Hereafter we define this absence of mCherry^+^ leukemia cells in the blood as a state of remission.

As observed previously^33^, despite ongoing Dox treatment almost all mice succumbed within 6 months to immature, proliferative, disseminated relapse resembling the original leukemia (Fig. 1b, c and Supplementary Fig. 1a, b). Notably, relapsed leukemia was GFP^HIGH^ in 12 of 13 mice examined, with low PU.1 protein similar to the original untreated leukemia (Fig. 1d). Relapse leukemias readily transplanted secondary recipients but remained GFP+ and were again unresponsive to in vivo Dox treatment (Supplementary Fig. 1c–e). Cultured leukemia cells from relapsed mice remained GFP^HIGH^ in the presence or absence of Dox, indicating that relapse is unlikely to arise from mutations in tTA that reverse its Dox sensitivity (Fig. 1e). Although the precise molecular correlates of relapse remain unknown, our results suggest acquired genetic or epigenetic changes that re-instate stable and Dox-resistant PU.1 shRNA expression.

### AML246 differentiation into two distinct myeloid lineages in vivo

**Fig. 2:**
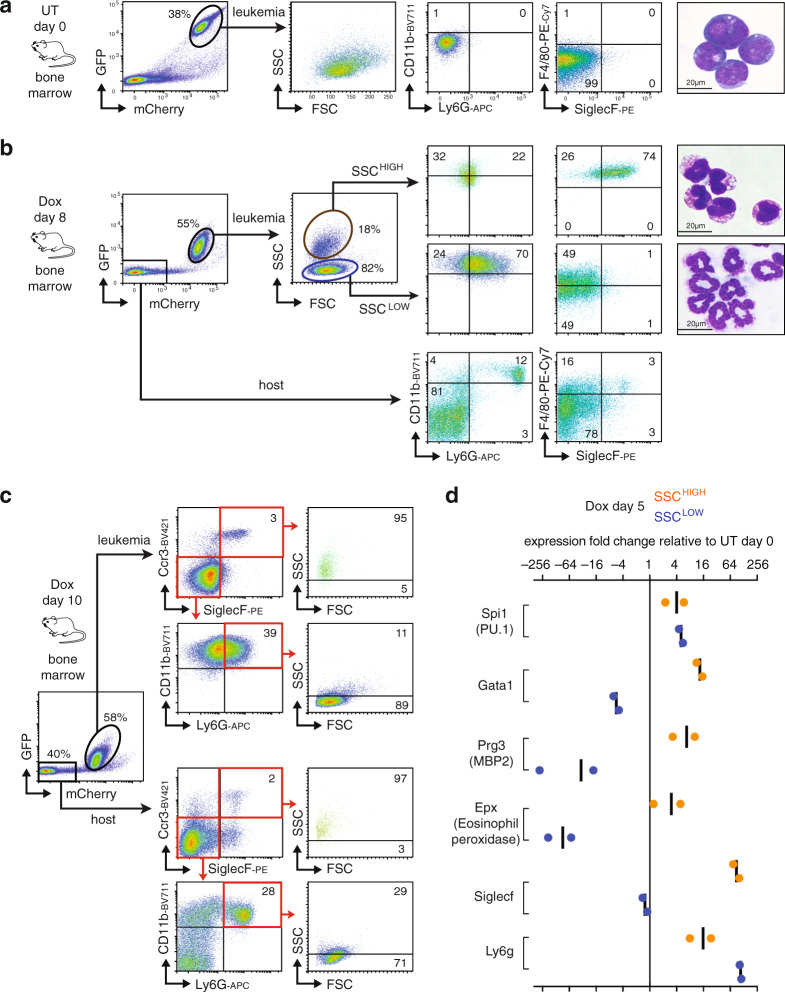
AML differentiation bifurcates into distinct mature myeloid lineages. **a**, **b** Flow cytometry of bone marrow from mice with established AML246-Cherry leukemia either untreated (**a**) or Dox treated for 8 days (**b**) and harvested on the same day to allow matched flow cytometry gating analysis. Data are representative of three independent experiments with similar results. Matched cytospins are shown for sorted mCherry^+^ cells from untreated mice and sorted mCherry^+^SSC^HIGH^ or mCherry^+^SSC^LOW^ AML-derived cells following 8 days Dox. **c** Immunophenotypic analysis comparing AML-derived (mCherry^+^) and host (mCherry^−^) cells within a representative AML246-Cherry leukemic mouse after 10 days Dox treatment. **d** mRNA expression by RT-qPCR of PU.1 and key neutrophil or eosinophil lineage genes in sorted mCherry^+^SSC^HIGH^ and mCherry^+^SSC^LOW^ AML-derived cells from 5 day Dox-treated mice, relative to mCherry^+^ AML cells from untreated mice. Bars indicate the average of the two samples shown.

Using flow cytometry we examined dynamic immunophenotypic changes of AML246-Cherry cells during early stages of in vivo Dox treatment. One week on Dox uniformly suppressed leukemia GFP expression in the blood, bone marrow, and spleen (Fig. 2a, b and Supplementary Fig. 2a). Intriguingly, Dox-induced differentiation resulted in the emergence of two leukemia-derived mature myeloid lineage sub-populations readily distinguishable from untreated leukemia and each other based on side scatter (SSC), immunophenotype, and morphology. The dominant AML-derived sub-population in the bone marrow after 8–10 days Dox had increased surface expression of the neutrophil marker Ly6G and a SSC^LOW^ profile similar to normal host CD11b^+^Ly6G^+^ neutrophils within the same samples (Fig. 2b, c and Supplementary Fig. 2b). These cells also had a ringed or segmented nucleus characteristic of neutrophils or their precursors (Fig. 2b and Supplementary Fig. 2c). After 8–10 days Dox we also invariably observed a distinct AML-derived sub-population comprising SSC^HIGH^F4/80^HIGH^ cells expressing the eosinophil surface markers SiglecF and Ccr3 at similar levels to normal host eosinophils within the same sample (Fig. 2b, c and Supplementary Fig. 2b). Indeed these cells had kidney-shaped nuclei and orange/red cytoplasmic staining reminiscent of eosinophils (Fig. 2b and Supplementary Fig. 2c). Hence, upon PU.1 restoration most AML246 cells undergo neutrophil lineage maturation but a minor population become eosinophil-like cells. Both mature populations were evident in the blood, bone marrow, and spleen (Fig. 2b, c and Supplementary Fig. 3a, b), and were also evident upon Dox treatment of mice harboring an independent, previously described AML246 clone^33^ (Supplementary Fig. 3c).

The mRNA yields from AML-derived SSC^HIGH^ cells sorted from Dox day 8 mice was consistently poor, potentially due to the high ribonuclease content of eosinophils. A briefer 5 day Dox treatment also caused bifurcation of mCherry^+^ cells into SSC^LOW^ and SSC^HIGH^ populations (Supplementary Fig. 3d), both yielding abundant mRNA. RT-qPCR analysis revealed similar PU.1 mRNA restoration in both Dox-treated populations relative to untreated leukemia (Fig. 2d). Notably, the AML-derived SSC^HIGH^ population showed specific induction of the essential eosinophil transcription factor Gata1 (see below) and the largely eosinophil-restricted genes Prg3 (MBP2) and Eosinophil peroxidase (Epx)^35^ (Fig. 2d). In contrast, these genes were repressed in AML-derived SSC^LOW^ cells, which instead preferentially induced the neutrophil marker Ly6G (Fig. 2d). These molecular data further suggest that acute PU.1 restoration leads to bifurcation of AML246 into differentiated AML-derived populations resembling mature neutrophils and eosinophils. Notably, neutrophil/eosinophil bipotential is a feature of some cells within the normal granulocyte/monocyte progenitor (GMP) population^36^, and is also supported by recent single cell transcriptome analysis^37^.

### Exclusive persistence of AML-derived eosinophil-like cells during remission

**Fig. 3:**
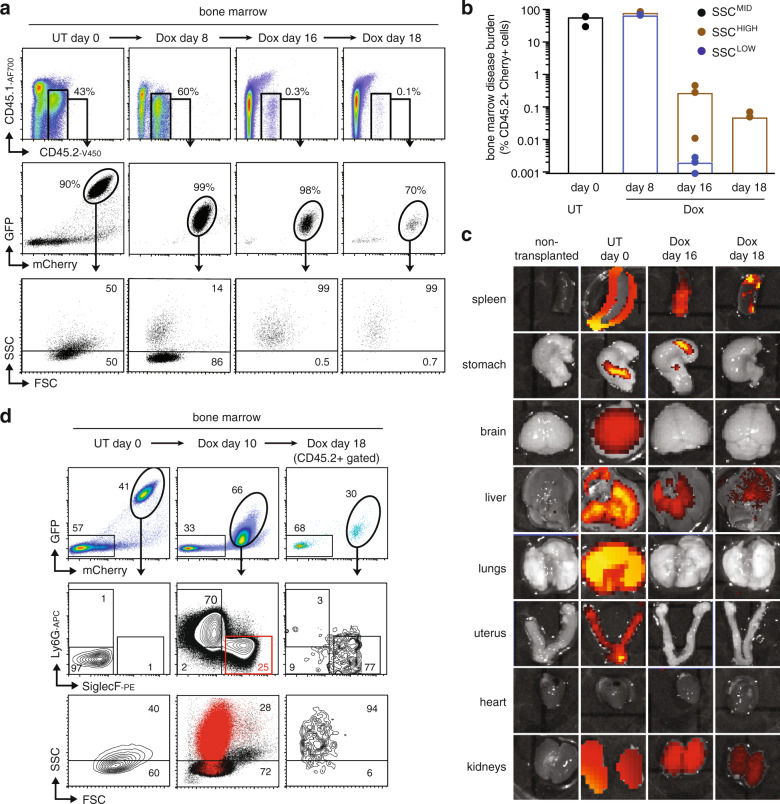
Persistence of AML-derived SSC^HIGH^ eosinophil-like cells during disease remission. **a** FSC/SSC profile of AML-derived cells harvested from the bone marrow of representative AML246-Cherry leukemic mice during an 18 day Dox treatment time course, showing viable AML-derived cells identified based on surface CD45.2 and mCherry. **b** Abundance and proportion of SSC^HIGH^ and SSC^LOW^ AML-derived cells during the 18 days Dox treatment time course (mean +/− standard error; *n* = 3 mice for UT day 0, Dox day 8 and 16; *n* = 2 mice for Dox day 18). **c** mCherry imaging by IVIS of organs from mice analyzed in a, either untreated or following 16–18 days Dox treatment. At each time point leukemic mouse organs were imaged alongside matched control organs from a non-transplanted *Rag1*^*−/−*^ mouse for signal calibration. **d** Flow cytometry analysis of AML-derived (mCherry^+^) cells from representative mice showing leukemia bifurcation into GFP^LOW^SSC^LOW^Ly6G^+^ neutrophil-like cells and GFP^LOW^SSC^HIGH^SiglecF^+^ eosinophil-like cells at Dox day 10, and relative persistence of AML-derived eosinophil-like cells at Dox day 18. Cells from the red SiglecF^+^ gate in the middle plot are shown in red in the SSC/FSC plot below.

We transplanted AML246-Cherry cells into *CD45.1* (*Ly5.1* or *Ptprc*^*a*^) recipient mice to allow improved resolution of leukemia-derived cells during regression and remission based on coincident mCherry fluorescence and surface CD45.2 (Ly5.2 or Ptprc^b^) expression. This approach revealed that following disease establishment and subsequent 16–18 days of Dox treatment, when leukemia cells were completely cleared from the blood, residual CD45.2^+^mCherry^+^ cells comprised only ~0.1% of the bone marrow and spleen (Fig. 3a, b and Supplementary Fig. 4a). Notably, during day 16–18 of Dox-induced remission the AML-derived SSC^LOW^ neutrophil-like population dominant at day 8 was no longer present (Fig. 3a, b and Supplementary Fig. 4a), consistent with the short lifespan of normal neutrophils^1,38^. Instead, residual CD45.2^+^mCherry^+^GFP^LOW^ AML-derived cells in the bone marrow and spleen were exclusively SSC^HIGH^ (Fig. 3a, b and Supplementary Fig. 4a). Organ imaging by IVIS identified persistent mCherry signal in the spleen after 16–18 days Dox treatment, consistent with splenocyte flow cytometry (Fig. 3c and Supplementary Fig. 4b). Notably, mCherry fluorescence was also reproducibly detected in the liver and kidneys at this time point, while other organs were disease-free (Fig. 3c and Supplementary Fig. 4b). Residual CD45.2^+^mCherry^+^ cells in the bone marrow, spleen, and liver of 16–18 days Dox-treated mice were SSC^HIGH^ and expressed eosinophil markers (Fig. 3d and Supplementary Fig. 4c). Together, this data indicate that mature AML-derived SSC^LOW^Ly6G^+^ neutrophils are cleared within 2 weeks, leaving persistent AML-derived mature eosinophil-like cells in the bone marrow, spleen, liver, and kidneys.

### High resolution in vivo analysis of early leukemia relapse

**Fig. 4:**
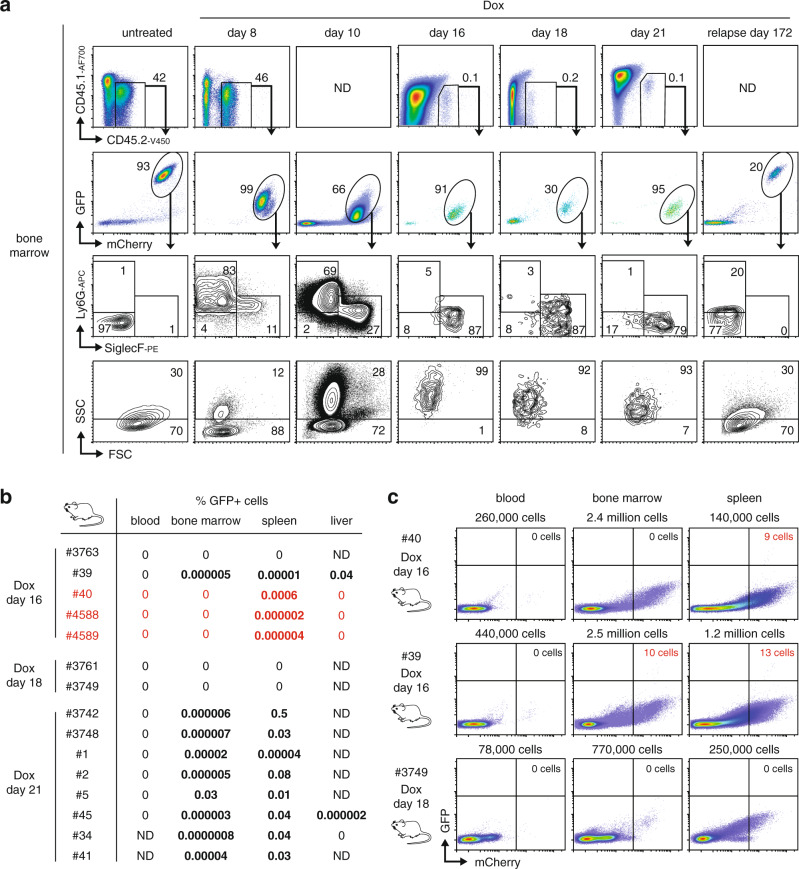
Analysis of early leukemia relapse in vivo. **a** Flow cytometry analysis of AML-derived (mCherry^+^) cells from representative mice harvested at various Dox treatment timepoints and at relapse. ND, not determined (host mouse was CD45.2^+^). **b** Summary of the distribution of mCherry^+^GFP^+^ early relapse cells detected in mice following the indicated Dox treatments. Mice with extramedullary relapse are shown in red. ND not determined. **c** Flow cytometry of blood, bone marrow, and spleen detecting organ-specific GFP^+^ leukemia relapse (red labeled quadrant) in representative AML246-Cherry leukemic mice following 16–18 days Dox treatment. The GFP threshold is set high to avoid the mCherry “smear” associated with leukemia cell death and host-mediated clearance, and to identify genuine GFP^HIGH^ early relapse.

Analysis of leukemic mouse bone marrow across a Dox treatment time course showed initial leukemia neutrophil/eosinophil bifurcation, followed by specific persistence of rare AML-derived eosinophil-like cells during remission (Fig. 4a). Almost all mice on long term Dox treatment relapsed with immature GFP^HIGH^ leukemia resembling untreated disease (Fig. 4a), indicating GFP may provide a sensitive marker of early relapse. Dox-resistant mCherry^+^GFP^HIGH^ cells were difficult to distinguish from the GFP^MID^ leukemia bulk at early Dox time points, but in some mice were clearly evident from Dox day 10 onwards as the leukemia bulk became GFP^LOW^ (Supplementary Table 2). Using deep flow cytometry to assess millions of cells per sample, we identified rare (often around 1 in 100,000 cells) mCherry^+^GFP^HIGH^ cells in at least one organ in 4 out of 7 mice examined at 16–18 days of Dox, and in all eight mice at 21 days of Dox (Fig. 4b, c). Unlike residual mCherry^+^GFP^LOW^ eosinophil-like cells in the same samples, these rare mCherry^+^GFP^HIGH^ cells had an immature immunophenotype and SSC^MID^ profile resembling the original leukemia, indicating early relapse associated with PU.1 knockdown (Supplementary Fig. 4c, d). For two Dox day 21 mice with rare mCherry^+^GFP^HIGH^ cells in the bone marrow and spleen (#3742, #3748) multi-organ mCherry imaging detected disease only in the spleen, liver, and kidneys, indicating early relapse without widespread leukemia dissemination (Supplementary Fig. 4b, e). Three 16 day Dox treated mice (#40, #4588, and #4589) harbored GFP^HIGH^ cells in the spleen but not the bone marrow or other organs (Fig. 4b, c and Supplementary Fig. 4b, c). These results are consistent with a potential extramedullary origin of leukemia relapse in some mice.

### Gata1 knockdown selectively prevents eosinophilic leukemia maturation

Our results suggested two potential sources of leukemia relapse in Dox-treated mice: (1) rare immature AML cells with up-front Dox resistance that prevents differentiation, or (2) rare mature leukemia-derived cells that acquire Dox resistance then revert to an immature proliferative state. To test the latter possibility we flow sorted mature mCherry^+^GFP^LOW^SSC^LOW^Ly6G^+^ neutrophil-like cells or mature mCherry^+^GFP^LOW^SSC^HIGH^ SiglecF^+^ eosinophil-like cells from day 5 or day 8 Dox-treated mice. Unfortunately these mature AML-derived cells rapidly lost viability in culture, precluding ex vivo reversion analysis. Transplant of 300,000 sorted mature AML-derived cells into untreated or Dox-treated mice did not cause leukemia development, however this may reflect an inability of mature blood cells to home and engraft the bone marrow upon intravenous transplantation (see “Discussion” section).

To avoid the inherent limitations of transplant-based leukemogenesis assays we instead adopted a genetic approach. We reasoned if the therapy-induced persistent population of mature AML246-derived eosinophils contributes to disease relapse, blockade of eosinophil lineage maturation may reduce relapse rates. To assess this we initally targeted Gata1, a DNA-binding zinc finger transcription factor. Gata1 plays an important role in megakaryocyte and erythroid lineage development, and is also essential for development of eosinophils, basophils, and dendritic cells^39^. However it is well established that the GMPs that give rise to the neutrophil and monocyte lineages are unaffected by Gata1 loss, and Gata1 is dispensable during maturation of these lineages^35,40–42^. In contrast, Gata1 induction in a subset of GMPs is essential for triggering eosinophil lineage commitment, and Gata1 expression remains high and indispensable throughout eosinophil development (Supplementary Fig. 5a, b)^35,36,42^. Given that AML246 is arrested at a GMP-like stage with neutrophil/eosinophil bipotential, we reasoned that Gata1 loss may block AML246 eosinophil differentiation while allowing neutrophil differentiation.

**Fig. 5:**
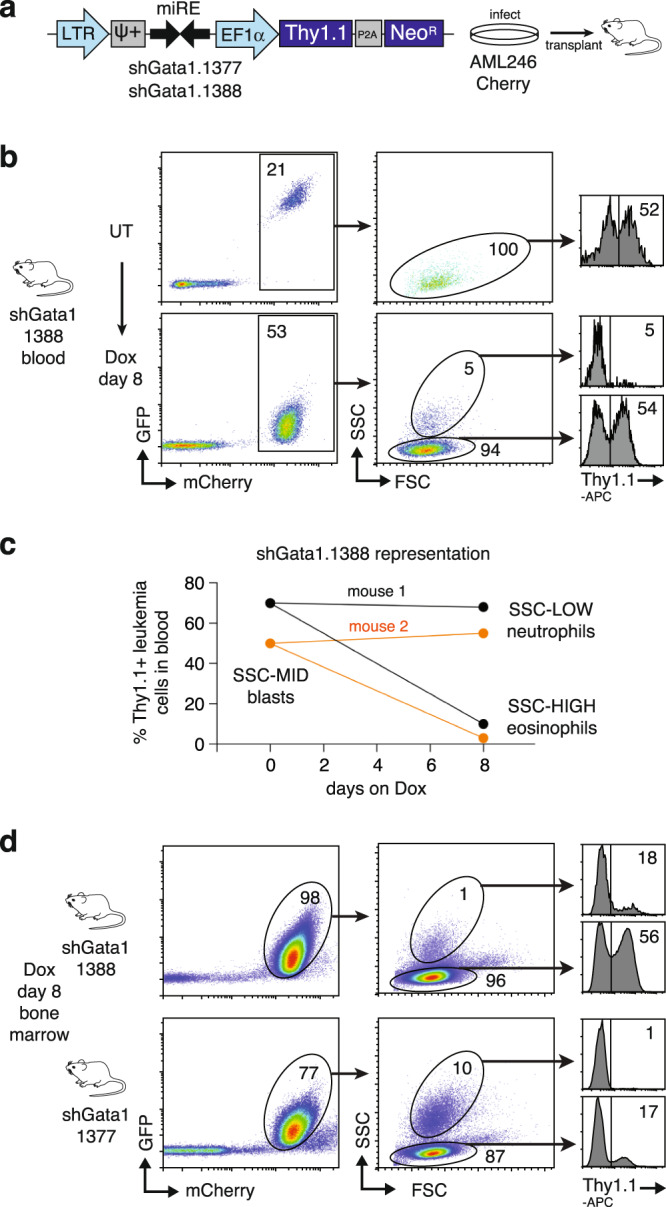
Gata1 knockdown prevents eosinophilic maturation of AML246. **a** Schematic of the LETN shRNA vector co-expressing the surface marker Thy1.1. **b** Flow cytometry of blood from a representative mouse transplanted with AML246-Cherry stably transduced with LETN-shGata1.1388, either untreated or after 8 days Dox. **c** Proportion of Thy1.1^+^ (shGata1.1388 expressing) leukemia cells within each indicated population in the blood of two representative mice before and after 8 days on Dox. **d** Flow cytometry of bone marrow from representative mice harboring AML246-Cherry transduced with LETN-shGata1.1388 or shGata1.1377 following 8 days of Dox treatment.

To inhibit Gata1 we transduced cultured AML246-Cherry cells with vectors stably co-expressing the cell surface marker Thy1.1 along with a microRNA-based Gata1 shRNA (Fig. 5a). Notably, Thy1.1^+^ cell representation was maintained following leukemia transplant and engraftment in recipient mice, indicating that Gata1 knockdown does not affect immature leukemia cells as predicted (Fig. 5b). However upon Dox-induced leukemia maturation in vivo, whereas the proportion of Thy1.1^+^ cells within the AML-derived neutrophil population remained steady, in AML-derived eosinophil-like cells from the same mice it fell dramatically (Fig. 5c, d). Hence, consistent with its role in normal GMPs, Gata1 suppression in vivo has no discernible effect on the proliferation of immature (GMP-like) AML246 or its neutrophil derivatives but blocks leukemia-derived eosinophil maturation.

### Deleting Gata1 or Xbp1 in leukemia prevents eosinophilic maturation

**Fig. 6:**
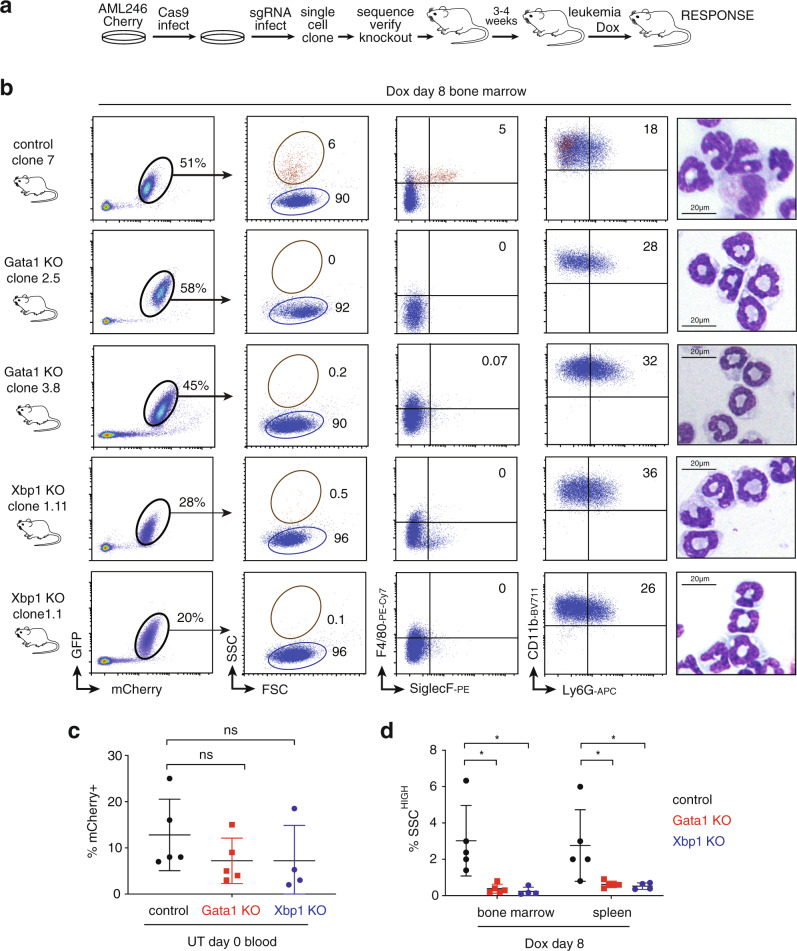
Gata1 or Xbp1 ablation prevents AML246 maturation into SSC^HIGH^ eosinophil-like cells. **a** Strategy for CRISPR/Cas9-mediated gene knockout in AML246-Cherry cells. **b** Flow cytometry of bone marrow from representative *Rag1*^*−/−*^ mice transplanted with the indicated AML246 single cell clones, allowed to develop disease, and then treated with Dox for 8 days (Table S3). A control sgRNA clone (top row) is compared to two independent AML246-Cherry Gata1 KO clones (second and third rows) and two independent AML246-Cherry Xbp1 KO clones (fourth and fifth rows). Data are representative of two independent experiments with similar results. Cytospins of sorted mCherry^+^ AML-derived cells are also shown. **c** Proportion of mCherry^+^ AML cells in the peripheral blood of mice transplanted with AML246-Cherry control sgRNA clones (black), Gata1 knockout clones (red), or Xbp1 knockout clones (blue) upon disease establishment when Dox treatment commenced. Mean +/− SD. *p* = 0.21 and *p* = 0.31 for Gata1 and Xbp1 ko clones respectively versus control, unpaired two-sided Student’s *t*-test. *n* = 5 mice for control sgRNA clones and Gata1 knockout clones, and *n* = 4 mice for Xbp1 knockout clones over two independent experiments. **d** Proportion of mCherry^+^ AML cells that were also SSC^HIGH^ in the bone marrow and spleen of mice from **c** following 8 days Dox treatment. Mean +/− SD. Gata1 versus control *p = 0.017 and *p* = 0.047 for bone marrow and spleen respectively, Xbp1 versus control *p = 0.019 and *p* = 0.037 respectively, unpaired two-sided Student’s *t*-test. Mouse numbers as for 6c.

We then used lentiviral CRISPR/Cas9 to ablate Gata1 in AML246-Cherry, generating several clones derived from single cells with unique biallelic Gata1 frameshift mutations that disable both full length Gata1 protein and the shorter Gata1s isoform (Fig. 6a and Supplementary Fig. 5c, d)^43^. In culture, Gata1-deficient clones were immunophenotypically indistinguishable from parental AML246 and control clones, with similar proliferation rates and Dox responses (Supplementary Fig. 6a, b). Serial dilution transplantation assays verified similar leukemogenic capacity of untreated Gata1-deficient and control clones (Supplementary Fig. 7a; and see below), and all resulting leukemias were immunophenotypically immature (Supplementary Fig. 7b). The immediate in vivo Dox response of Gata1-deficient and control leukemias was similar, with acute GFP repression and regression within 2 weeks (Supplementary Fig. 7c–e). In mice harboring control sgRNA leukemia clones, acute Dox treatment triggered neutrophil/eosinophil bifurcation as for parental AML246-Cherry (Fig. 6b–d). In contrast, Dox-induced differentiation of Gata1 knockout leukemia failed to produce mCherry^+^SSC^HIGH^SiglecF^+^ eosinophil-like cells (Fig. 6b–d and Supplementary Fig. 7f). Instead it exclusively produced neutrophil-like cells, consistent across multiple independent clones (Fig. 6b, d).

As an additional strategy to block eosinophil maturation of AML246 we genetically ablated Xbp1, another transcription factor selectively required for eosinophil differentiation^44^. It is well established that Xbp1 knockout in mice does not affect GMPs or neutrophils, but prevents eosinophil differentiation beyond the eosinophil precursor (EoP) stage following lineage commitment^44^ (Supplementary Fig. 8). In keeping with this, untreated, immature Xbp1 knockout clones were indistinguishable from Gata1-deficient or control clones in culture and upon transplant into recipient mice (Supplementary Figs. 6a, b and 7b, c). However upon Dox-induced maturation in vivo, Xbp1-deficient AML246 clones did not differentiate into the eosinophil lineage but instead exclusively produced neutrophils (Fig. 6b, d). Hence, Gata1 and Xbp1 are dispensable in immature AML246 leukemia cells but essential for leukemia maturation into the eosinophil lineage following in vivo differentiation therapy.

### Blocking eosinophil maturation can prevent AML relapse

**Fig. 7:**
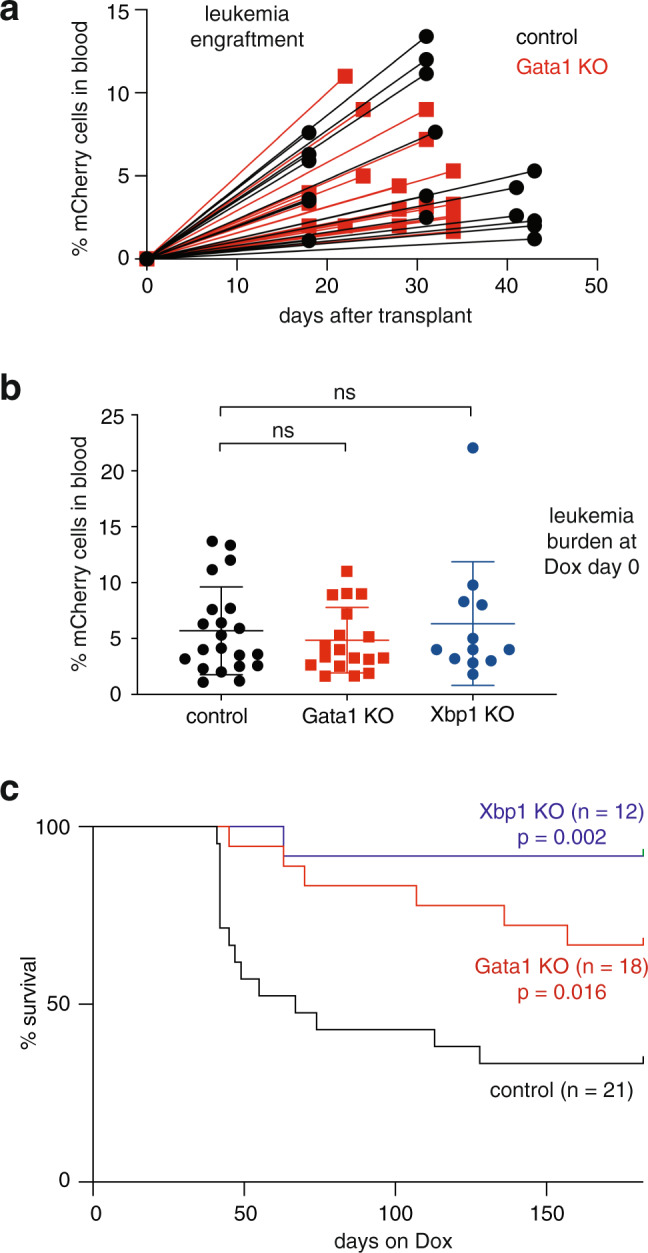
Eosinophil lineage ablation can prevent AML relapse following differentiation therapy. **a** Blood leukemia burden indicating engraftment of control and Gata1 knockout AML246 clones in recipient mice following intravenous transplant of 1 million leukemia cells. **b** Proportion of mCherry^+^ AML cells in the peripheral blood of mice transplanted with AML246-Cherry cells from control sgRNA clones (black), Gata1 knockout clones (red), or Xbp1 knockout clones (blue). Bleeds were taken at the time of established disease, when Dox treatment was initiated for the survival analysis shown in **c**. *n* = 21, 18, and 12 mice per group respectively over two independent experiments. Mean +/− SD. Relative to control, Gata1 KO *p* = 0.46 and Xbp1 KO *p* = 0.7, unpaired two-sided Student’s *t*-test. **c** Kaplan–Meier survival analysis of mice from **b** over 6 months of sustained Dox treatment. Relative to control, Gata1 KO *p* = 0.016 and Xbp1 KO *p* = 0.002, Mantel-Cox test. *n* = 21, 18, and 12 mice per group respectively. Source data are provided as a Source Data file.

To examine the impact of eosinophil lineage blockade on long term differentiation therapy outcome, we transplanted mouse cohorts with control, Gata1 knockout, or Xbp1 knockout AML246-Cherry (Supplementary Table 3). Consistent with earlier transplants (Supplementary Fig. 7a), control and Gata1 knockout cells engrafted recipient mice with comparable kinetics and caused overt leukemia within a few weeks (Fig. 7a). After ensuring each group had similar peripheral blood disease burden, Dox treatment was initiated (Fig. 7b). Over the course of 6 months Dox treatment, several mice harboring Gata1- or Xbp1-deficient leukemia succumbed to relapse, however overall survival was significantly improved relative to controls (Fig. 7c; *p* = 0.016 or 0.002, Mantel-Cox test). Only 6 of 18 mice harboring Gata1 knockout leukemia and 1 of 12 mice with Xbp1 knockout leukemia relapsed within a 6-month period on Dox, compared with 14 of 21 control mice (Fig. 7c). Hence preventing eosinophil lineage maturation leads to leukemia eradication in a significant proportion of mice in this differentiation therapy model.

### Human AML-derived eosinophil-like cells can revert to an immature proliferative state

**Fig. 8:**
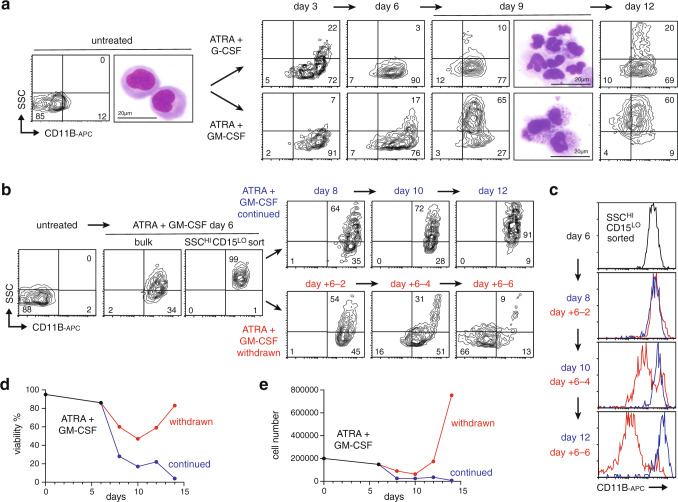
De-differentiation of human AML-derived eosinophil-like cells upon therapy cessation. **a** SSC/CD11B profiles of HT93 cells during ATRA treatment combined with G-CSF (upper panels) or GM-CSF (lower panels). Representative cytospins are shown for untreated (UT) and ATRA day 9 treatments. This experiment was performed once. **b** SSC/CD11B profiles of SSC^HIGH^CD11B^+^CD15^–^ cells sorted from ATRA + GM-CSF cultures then either maintained on treatment (upper panels) or following ATRA/GM-CSF withdrawal (lower panels). **c** Surface CD11B profile of cells from **b**, showing progressive return to an immature state following treatment withdrawal. **d**, **e** Viability (**d**) and cell count (**e**) of HT93 cells from **b**, showing rebound following treatment withdrawal.

The human t(15;17) APL cell line HT93 has a reported capacity to differentiate into neutrophils or eosinophils in response to the clinical differentiation therapy ATRA^45^, suggesting a lineage bipotency similar to AML246. Indeed, consistent with previous reports^45^, we found that combining ATRA with granulocyte colony-stimulating factor (G-CSF) triggered HT93 differentiation mainly into SSC^LOW^CD11B^+^CD15^+^ cells with multilobed nuclei characteristic of neutrophils (Fig. 8a and Supplementary Fig. 9a). In contrast, combining ATRA with granulocyte-macrophage CSF (GM-CSF) preferentially produced SSC^HIGH^CD11B^+^CD15^–^ cells with densely granulated cytoplasm resembling eosinophils (Fig. 8a and Supplementary Fig. 9a). Notably, sustained ATRA/GM-CSF treatment of mature HT93 cells led to elimination of the major leukemia-derived neutrophil population, resulting in exclusive persistence of human AML-derived eosinophil-like cells (Fig. 8a and Supplementary Fig. 9a). These results recapitulate mouse AML246 lineage dynamics in vivo (Fig. 3), providing further evidence for relative persistence of AML-derived eosinophil-like cells over AML-derived neutrophils.

To determine whether human AML-derived eosinophil-like cells could revert to an immature proliferative state we sorted the SSC^HIGH^CD11B^+^CD15^–^ eosinophil population from 6 day ATRA/GM-CSF treated HT93 cultures, then withdrew ATRA/GM-CSF. Treatment cessation resulted in progressive reductions in SSC and surface CD11B across the entire cell population, which returned to its original immature state within one week accompanied by a rebound in viability and proliferation (Fig. 8b–e and Supplementary Fig. 9b, c). These results demonstrate *en masse* de-differentiation of human leukemia-derived eosinophils upon loss of the differentiation stimulus. Notably this maturation state plasticity of HT93 cells occurs in the context of wild type *TP53*^33^.

## Discussion

Using a mouse model we have studied AML differentiation therapy response and relapse at a resolution not possible in AML patients, identifying an unforeseen sub-lineage of mature leukemia-derived cells that persists during remission and contributes to disease relapse. While certain features of this model (e.g., eosinophil lineage maturation) may be somewhat unique to AML246, our findings illustrate concepts of broad relevance to differentiation therapy response across AML subtypes. Our results suggest that immature AML relapse can originate from a persistent non-neutrophil lineage of mature leukemia cells, in this case leukemia-derived eosinophils, which implicates maturation state plasticity as a mechanism of differentiation therapy resistance and subsequent relapse. Consistent with this, we also observe remarkable phenotypic plasticity of human AML-derived eosinophil-like cells upon ATRA exposure and withdrawal in culture. Although we have not directly observed leukemia maturation state plasticity in AML patients, our results provide impetus for longitudinal studies of clinical samples using emerging single cell technologies (see below).

Our results highlight potential drawbacks of traditional transplant-based leukemogenesis assays, which may ascribe “leukemia stem cell” (LSC) activity to the most immature leukemia cells in a tumor population based simply on preferential engraftment capacity. To circumvent inherent transplantation bias we instead adopted a novel genetic approach, uncovering a mature and non-LSC origin of leukemia relapse. Our results add to several recent studies describing novel non-LSC mechanisms of resistance to cytotoxic AML therapy, which include therapy-induced metabolic adaptation or outgrowth of pre-existing therapy-resistant leukemia sub-populations^46–48^.

Therapy-induced in vivo bifurcation of bulk AML246 into the neutrophil and eosinophil lineages is maintained even after single cell cloning, likely reflecting an intrinsic bipotency of the myeloid progenitor state from which the leukemia is derived. Although diverse leukemia-derived lineages are evident in some AML patients, therapy-induced maturation is often broadly classified as “granulocytic” based on a spike in blood neutrophil counts during induction. Similarly, the initial wave of AML246 differentiation in mice is dominated by leukemia-derived neutrophils, with a minor subpopulation of leukemia-derived eosinophils only becoming dominant after neutrophil clearance. Rapid therapy-induced clearance of AML-derived neutrophils reflects the high turnover rate of normal neutrophils^38^, and persistence of leukemia-derived eosinophils in certain tissues may reflect the slower turnover of this lineage^49,50^. Indeed in an intriguing parallel to AML246, abundant leukemia-derived eosinophils were identified in the bone marrow of an enasidenib-treated patient months after treatment^51^. Other mature leukemia-derived myeloid lineages including monocytes/macrophages may play an important role in therapy-induced residual disease in certain AML subtypes, but this awaits further investigation. In any case, by showing that a minor lineage of persistent mature leukemia-derived cells can resist differentiation therapy and seed relapse, our results highlight the importance of molecular monitoring of residual disease in addition to traditional morphological or immunophenotypic measures.

Our results suggest two major modes of AML246 relapse in Dox treated mice. In some mice rare immature and Dox-resistant GFP^HIGH^ cells are detected within 10 days on Dox, consistent with pre-existing genetic or epigenetic alterations that maintain GFP-linked PU.1 shRNA expression. Such up-front Dox resistance of immature leukemia cells may occur in both parental AML246 and its Gata1-deficient or Xbp1-deficient derivatives, and may contribute to the low frequency relapses observed with eosinophil-incapable leukemias. Conversely, several observations indicate that relapse in a significant proportion of mice originates from mature leukemia-derived cells. Firstly, deep flow cytometry of multiple organs after 7–10 days of Dox treatment shows synchronous GFP repression across the entire leukemia population in most mice, suggesting uniform PU.1 restoration and *en masse* differentiation. Secondly, after 16–21 days of Dox treatment, measurable residual disease in several mice exclusively comprises mature eosinophil-like cells. Thirdly, in some cases we observe isolated extramedullary relapse, potentially originating from mature leukemia-derived cells that preferentially reside in peripheral organs rather than the bone marrow. Finally, blockade of eosinophilic leukemia maturation by ablating Gata1 or Xbp1 prevents relapse in a significant proportion of mice. Neither gene is essential in GMPs, but Gata1 is required for GMP commitment to the eosinophil lineage^40^ and Xbp1 for eosinophil progenitor (EoP) survival^44^. Hence our results suggest that in some mice immature AML cells must commit to the eosinophil lineage in order to persist and seed relapse. The extent of AML246-derived eosinophil maturation compatible with subsequent de-differentiation and relapse remains unclear. It is plausible that the striking phenotypic maturity of persistent AML246-derived eosinophils (including surface Ccr3 expression) promotes tissue residency^52^, creating a potential source of the extramedullary relapse observed in some mice. Notably, we also find that the SSC^HIGH^ and heavily granular human eosinophil-like cells derived from ATRA-treated HT93 cultures can revert to an immature state, demonstrating remarkable phenotypic plasticity of this lineage.

Rapid in vivo clearance of leukemia-derived neutrophils in our AML model provides rationale for combining differentiation therapy with agents that promote neutrophilic AML differentiation. Cytokines such as G-CSF can instruct lineage choice^53^, and may influence leukemia fate if co-administered with differentiation therapy. Our data also show that eliminating a mature leukemia-derived lineage can reduce relapse, providing proof-of-principle for therapeutic targeting of persistent leukemia-derived mature cells. This may be achieved by blocking recruitment into organs, depleting lineage-supporting cytokines, or exploiting other lineage-specific vulnerabilities. While immune-based lineage depletion is clinically effective in B lineage malignancies based on restricted expression of CD19 and CD20, effective AML depletion therapies remain elusive because immature AML blasts share surface antigens with normal myeloid progenitors essential for survival^54^. AML differentiation therapy may however facilitate targeting of residual disease by inducing surface markers specific to non-essential or rapidly replenishable mature myeloid lineages. Analogous to Gata1 or Xbp1 knockout in AML246, such therapies may be ineffective at diagnosis and beneficial only after inducing AML maturation.

Rational therapeutic targeting of mature AML-derived cells in patients will rely on understanding the lineage potential of individual leukemias, which may vary depending on mutation profile and arrested position within the myeloid progenitor hierarchy^55^. Unbiased tracking of multilineage differentiation and high resolution phenotyping of residual disease now appears feasible in AML patient samples using single cell technologies that match surface marker or RNA-seq profiling with genetic mutation status^55,56^. Detailed residual disease phenotyping in AML patients during differentiation therapy may inform combination therapies designed to prevent or eradicate persistent mature AML sublineages and improve overall outcomes.

## Methods

### Animal studies

All mouse experiments including AML246 treatment studies were approved by the Alfred Research Alliance Animal Ethics Committee. Mice were housed with a 12 h light/dark cycle and standard air conditioned ambient temperature and humidity. Doxycycline (Sigma-Aldrich, St Louis, MO) was administered in the diet at 600 mg/kg food (Specialty Feeds, Glen Forrest, Western Australia).

### Mouse AML transplantation

To propagate leukemias, primary splenocytes or bone marrow cells from leukemic mice (predominantly leukemia cells) were transplanted by tail vein injection into immunocompromised *Rag1*^−/−^ recipient mice (~10^6^ cells/mouse). Mice transplanted with AML246 generally developed signs of leukemia after 4–6 weeks including loss of activity or weight, palpable splenomegaly, and elevated peripheral blood white cell counts. For in vivo PU.1 restoration experiments Dox treatment was commenced upon detection of ~2–10% mCherry^+^GFP^+^ tumor cells in peripheral blood. Mice were deemed to have relapsed when emergent mCherry^+^ leukemia cells in the peripheral blood exceeded 10% despite ongoing Dox treatment. Mouse survival analysis was performed using GraphPad Prism 8 (GraphPad Software).

### IVIS imaging

mCherry fluorescence in mouse organs was imaged by IVIS Lumina III (PerkinElmer) with the emission filter set to 620 nm and excitation filter set to 580 nm. Organs from leukemic mice were always imaged alongside negative control organs (harvested from untransplanted *Rag1*^−/−^ or *CD45.1 Rag1*^−/−^ mice) using recommended settings (exposure time 0.5–60 seconds, medium binning, 10 cm field of view) to allow signal calibration. Following image capture, autofluorescence was removed using the Adaptive Fluorescent background subtraction tool.

### Mouse AML culture

AML246 cells and derivatives were cultured in IMDM supplemented with 10% FBS, penicillin (100 U/mL), streptomycin (100 mg/mL) and 10 ng/mL IL-3 (Peprotech). Single cell clones were generated by sorting single cells into 96-well plates using a FACS Influx (BD Biosciences, San Jose, CA) followed by culture for 3–4 weeks. One microgram per millimeter of Doxycycline (Dox; Sigma) treatments were refreshed weekly. For proliferation analysis 50,000 cells were seeded in 24-well plate. Untreated cells were split 1:4 at day 4, 8, and 12, while Dox-treated cells were split 1:2 at day 4 only. Cell counts were assessed by flow cytometry using SpheroAccuCount Blank Beads (Spherotech). For viability analysis, cells were resuspended in FACS buffer (PBS with 5% FCS) with 1 μg/mL Sytox blue (ThermoFisher). For DNA content analysis, cells were permeabilized and fixed using the Cytofix/Cytoperm kit (BD Biosciences), stained with DAPI (49,6-diamidino-2-phenylindole dihydrochloride) (Sigma-Aldrich), and assessed by flow cytometry.

### Human HT93 cell culture

The human APL cell line HT93 (obtained from the Cell Resource Center for Biomedical Research, Tohoku University, Japan) was authenticated at CellBank Australia. HT93 cells were cultured in serum-free StemPro-34 media with 1× StemPro-34 nutrient supplement (Gibco), penicillin (100 U/mL), streptomycin (100 mg/mL), and 1× l-glutamine (Gibco). Cells were treated with 1 μM ATRA (Sigma) in combination with 50 ng/mL rhG-CSF (Filgrastim; Hospira) or 10 ng/mL GM-CSF (Peprotech) as required.

### Retroviral and lentiviral vectors

To generate AML246-Cherry we transduced AML246 Clone 1 cells^33^ with MSCV-IRES-mCherry (also known as MICR) using standard retroviral packaging protocols. Of several mCherry^+^ single cell clones tested, the AML246-Cherry clone was selected based on uniformly high mCherry fluorescence. For CRISPR/Cas9 gene editing, AML246-Cherry cells were lentivirally transduced with pHRSIN-pSFFV-FLAG-NLS-Cas9-NLS-pSV40 blasticidin (a gift from Marian Burr and Paul Lehner, University of Cambridge) and transduced cells were positively selected for two weeks in 40 µg/mL blasticidin with medium replacement twice per week. AML246-Cherry-Cas9 cells were lentivirally transduced with ETN (Lenti-hU6-sgRNA.iT-EF1as-Thy1.1-P2A-Neo) vectors harboring sgRNA sequences targeting murine Gata1, Xbp1, or a negative control sgRNA (Supplementary Table 4). Several single Thy1.1^+^ cell clones were generated and target gene mutations were identified by Sanger sequencing PCR products amplified from genomic DNA and/or cDNA (Supplementary Table 4). For knockdown studies we used the LETN (MSCV-miRE-EF1as-Thy1.1-P2A-Neo) vector, which was generated by cloning the EF1as-Thy1.1-P2A-Neo cassette from ETN into LENC (MSCV-miRE-PGK-Neo-IRES-mCherry) digested with SalI/MluI to remove its PGK-Neo-IRES-mCherry cassette. Control Renilla shRNA or Gata1 shRNA sequences were generated by oligonucleotide annealing (sequences in Supplementary Table 5), and cloned into the XhoI/EcoRI sites of LETN.

### Flow cytometry and cytospins

Blood was collected by mandible bleed. For mouse leukemia analysis, single cell suspensions from bone marrow, spleen, peripheral blood, or liver were treated with red cell lysis buffer (15 mM NH_4_Cl, 1 mM KHCO_3_, 0.01 mM EDTA) and washed twice in FACS staining buffer (PBS supplemented with 5% FCS) before incubating with fluorochrome-conjugated antibodies. For all flow cytometry immunophenotyping of mouse and human leukemias, cells were incubated for 5 min with anti-CD16/CD32 (unlabeled, clone 2.4G2, WEHI), then for 30 min on ice with the following antibodies against mouse antigens: CD11b-BV711 or CD11b-PE (clone M1/70, eBioscience), Ly6G-APC (clone 1A8-Ly6G, eBioscience), F4/80-PE/Cy7 (clone BM8, eBioscience), Siglec-F-PE (clone E50-1440, BD Biosciences), Thy1.1-APC (clone OX-7, BioLegend), CD45.1-AF700 (clone A20, BD Biosciences), CD45.2-BV421, CD45.2-V450, or CD45.2-PE/Cy7 (clone 104, BD Biosciences). All antibodies were used at 1:400 dilution. Following centrifugation, cell pellets were resuspended in 300 μL FACS staining buffer with SYTOX^TM^ blue (Thermo Fisher Scientific, 5 mM solution). Flow cytometry of surface marker expression was performed on gated viable (SYTOX^TM^ blue or Fixable Live Dead Dye negative) cells using BD FACs LSRII and FACS-sorting was performed on an Influx (BD Biosciences, San Jose, CA). Data were analyzed using Flowlogic software (Inivai Technologies). AML cells were sorted with an Influx (BD Biosciences) with a 100 µm nozzle. For cytospins, 50,000–100,000 AML cells were spun onto SuperFrost Plus microscope slides (Menzel Gläser). Dried cytospins were methanol fixed and stained with May-Grünwald Giemsa, mounted, and imaged with the Aperio ScanScope XT microscope. Representative images were taken with Aperio ImageScope software v11.2 (Leica).

### Immunoblotting

Western blotting was performed using rabbit polyclonal (T-21) or mouse monoclonal anti-PU.1 antibody (C-3; both Santa Cruz Biotechnology), mouse monoclonal anti α-tubulin antibody (B-5-1-2, Sigma-Aldrich), and rabbit polyclonal anti-acetyl Histone H3 antibody (06-599, Millipore).

### RNA extraction and RT-PCR analysis

FACs sorting of AML-derived cells from the bone marrow or spleen of leukemia mice following 5 days on Dox was performed on an Influx (BD Biosciences, San Jose, CA). RNA extraction from 2 × 10^5^ to 5 × 10^5^ sorted cells was performed using the RNeasy Mini Kit (Qiagen) per manufacturer instructions, quantified by Nanodrop 1000 spectrophotometer, and stored at −80 °C. cDNA was generated using Superscript III first strand synthesis supermix kit (Invitrogen). On ice, up to 5 µg of total RNA (same concentration of RNA for each sample) was mixed with 1 µL random hexamer, 1 µL of annealing buffer and RNAse-free water (total volume 8 µL). This was incubated for 5 min at 65 °C then placed on ice for 1 min. On ice, 10 µL of 2× First-Strand Reaction Mix (Invitrogen) and 2 µL of SuperScriptTM III/RNaseOUTTM Enzyme Mix (Invitrogen) were added. The sample was incubated for 10 min at 25 °C then 50 min at 50 °C. The reaction was terminated by 5 min at 85 °C and stored at –20 °C. One microliter of cDNA was added to 5 µL Promega Master mix 2×, 2 µL of 3 µM primers (Table S4) and 2 µL of H_2_O (final volume 10 µL) in a 384 well plate. RT-PCR was performed using a LightCycler 480 (Roche) using protocol: 95 °C 3 min, (95 °C 15 s, 60 °C 30 s, 72 °C 30 s) × 45 cycles. Transcript levels relative to Rn18s (18S ribosomal RNA; Table S4) were quantified using the ΔΔCt method.

### Reporting summary

Further information on research design is available in the Nature Research Reporting Summary linked to this article.

## Supplementary information

Supplementary Information

Reporting summary

## Data Availability

The authors declare that the data supporting the findings of this study are available within the paper and its supplementary information files. All unique materials are readily available from the authors. Source data are provided with this paper.

## Source data

Source Data
