# Supplementary material for: Acute myeloid leukemia maturation lineage influences residual disease and relapse following differentiation therapy
Source: Nat Commun. 2021 Nov 11;12:6546. doi: 10.1038/s41467-021-26849-w (PMC8586014; doi:10.1038/s41467-021-26849-w)
Supplement: Supplementary file 1 — Supplementary Information [file 41467_2021_26849_MOESM1_ESM.pdf]

SUPPLEMENTARY INFORMATION

"Acute myeloid leukemia maturation lineage influences residual disease and relapse following differentiation therapy"

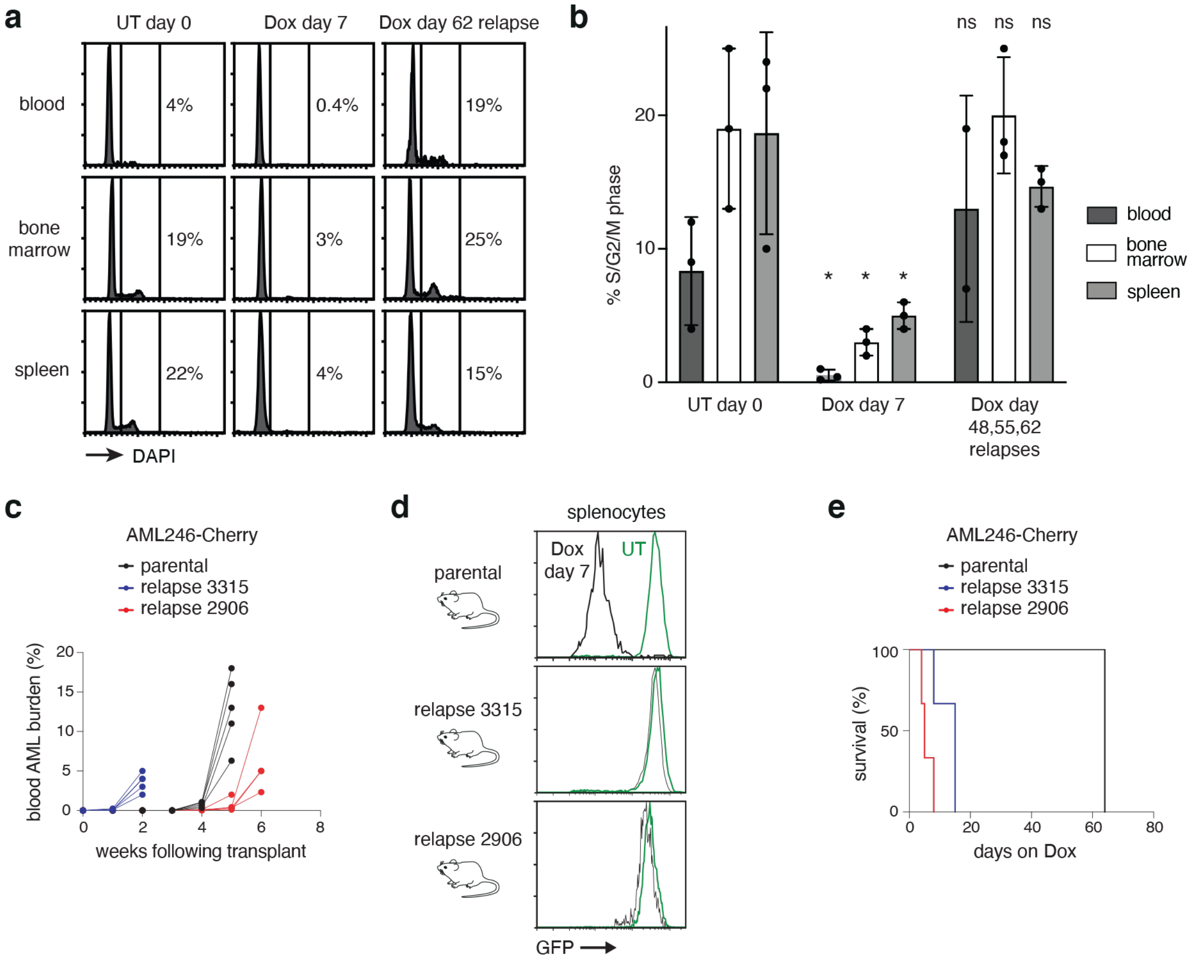

Supplementary Figure 1. Cell cycle and GFP analysis upon Dox-induced AML246 differentiation and relapse. (a) DAPI cell cycle analysis indicating the proportion of mCherry$^+$ cells in S/G2/M phases of the cell cycle in the blood, bone marrow, and spleen of representative untreated, Dox day 7 treated and Dox-treated relapsed mice. (b) Summary of the proportion of AML derived mCherry$^+$ cells in S/G2/M phase for each organ and time point. Mean +/– SD, n=3 mice per condition. Relative to untreated p=0.01 for bone marrow, 0.03 for blood and 0.04 for spleen, unpaired two-sided Student's t-test. (c) Proportion of mCherry$^+$ AML cells in the peripheral blood of untreated mice re-transplanted with AML cells from relapse mouse 3315 (blue), relapse mouse 2906 (red) or AML246-Cherry parental control cells (black) showing the rate of disease establishment. (d) GFP profile of AML cells in the blood of representative mice from each subgroup at the commencement of Dox treatment (untreated) and after 7 days of Dox treatment. (e) Kaplan-Meier survival analysis of mice from c over 2 months of sustained Dox treatment initiated upon disease establishment at day 0. p=0.017 for relapse 2906 or relapse 3315 versus parental control, Mantel-Cox test. Source data are provided as a Source Data file.

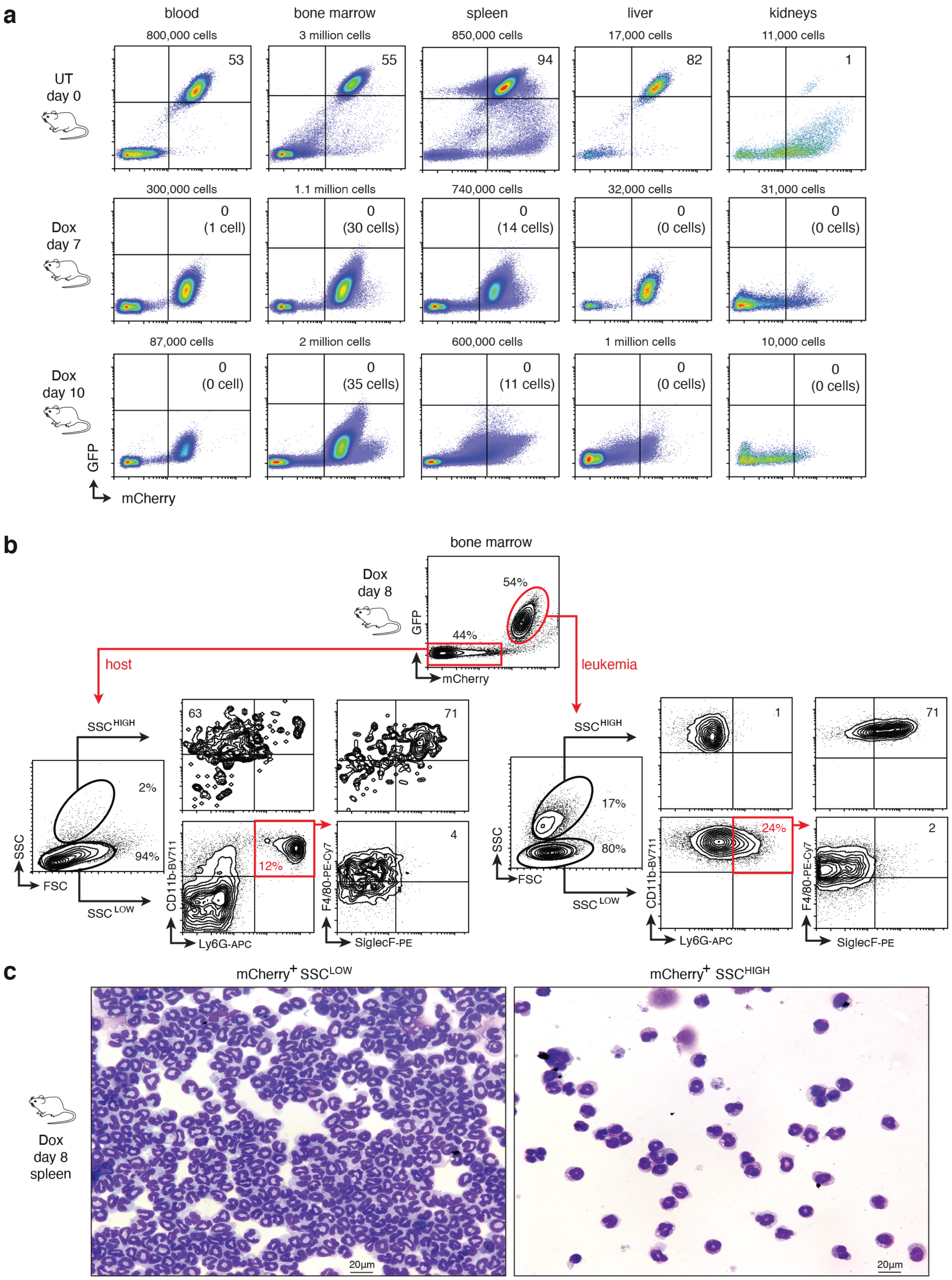

Supplementary Figure 2. Bifurcated AML differentiation in multiple organs following Dox treatment. (a) Flow cytometry analysis of mCherry and GFP in the blood, bone marrow, spleen, liver and kidneys of *Rag1*$^{-/-}$ mice with established AML246 that were either untreated or Dox treated as indicated. (b) Flow cytometry analysis of the bone marrow of the 8 day Dox treated mouse shown in Fig. 2b, comparing the immunophenotype of AML-derived (mCherry^{+}) SSC^{LOW}

and SSC^{HIGH} populations to host ($mCherry^{-}$) SSC^{LOW} and SSC^{HIGH} populations containing normal $CD11b^{+}Ly6G^{+}$ neutrophils and $F4/80^{HIGH}SiglecF^{+}$ eosinophils respectively. (c) Wide field cytospins of sorted $mCherry^{+}SSC^{LOW}$ and $mCherry^{+}SSC^{HIGH}$ cells from the spleen of the 8 day Dox treated mouse shown in Fig. 2b. Data are representative of 2 independent experiments with similar results.

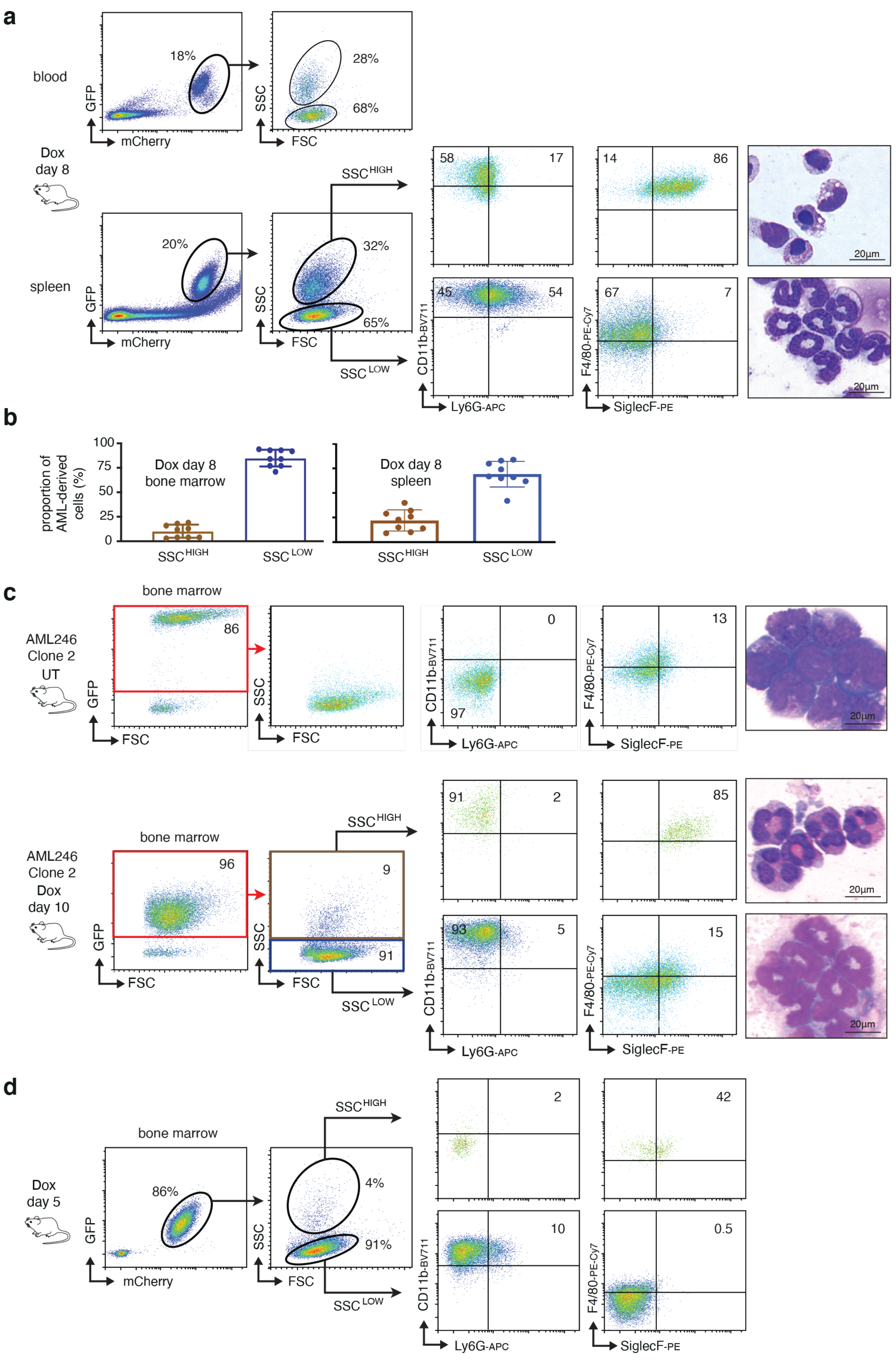

Supplementary Figure 3. Dox-induced AML246 bifurcation in several *in vivo* contexts. (a) Flow cytometry analysis of blood and spleen from the 8 day Dox treated mouse shown in Fig. 2b.

Data are representative of 3 independent experiments with similar results. (b) Quantitative analysis showing reproducible AML246 bifurcation in the bone marrow and spleen. Mean +/– SD, n=9 mice over 3 independent experiments. (c) Analysis of bone marrow from mice transplanted with AML246 Clone 2 (noting that AML246-Cherry was derived from AML246 Clone 1[1]). Data are representative of 2 independent mice with similar results from a single experiment. (d) Flow cytometry analysis of *Rag1*$^{-/-}$ mice with established AML246 treated with Dox for 5 days, showing early bifurcation of AML-derived cells into SSC^{HIGH} and SSC^{LOW} populations.

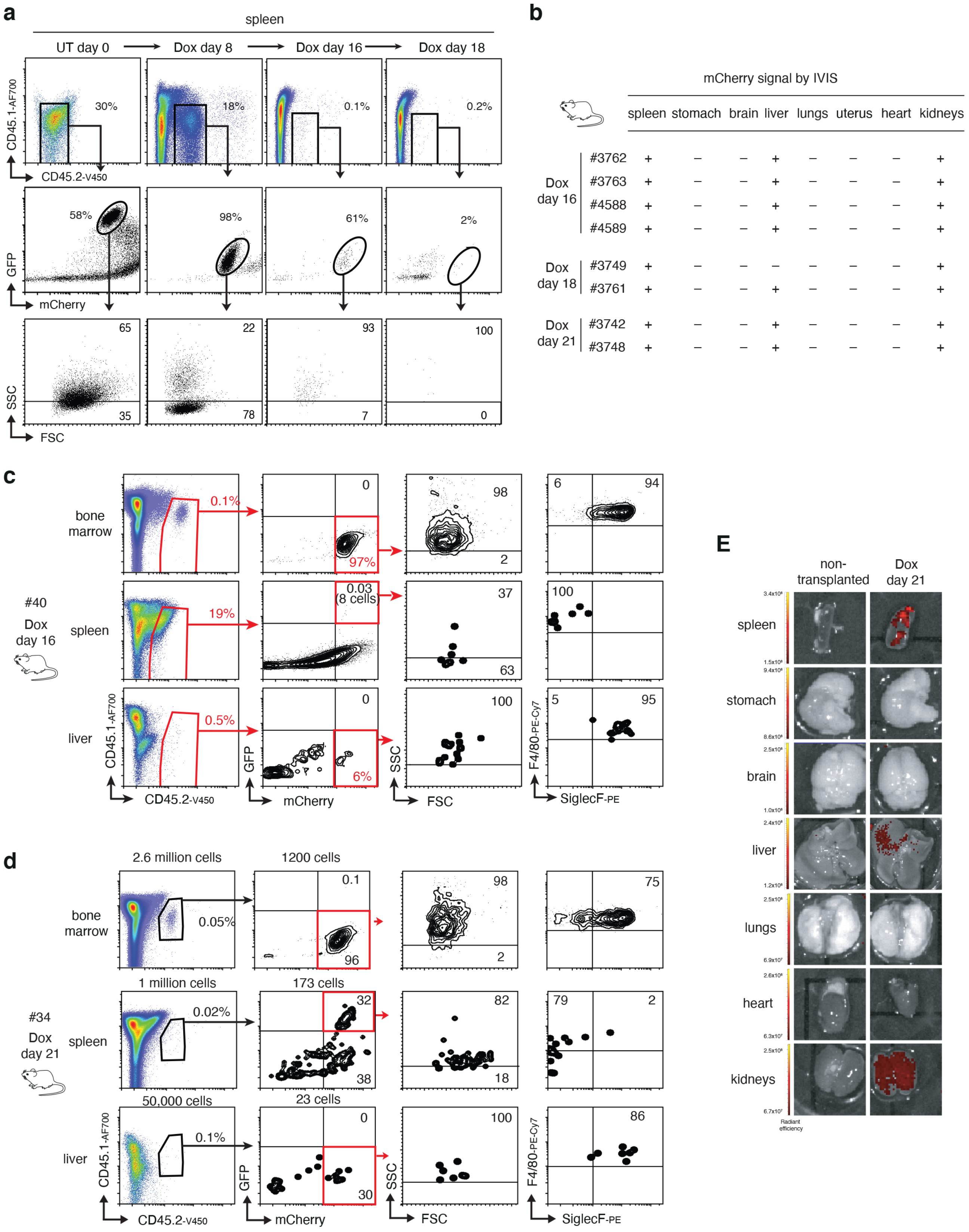

Supplementary Figure 4. Analysis of 16-21 day Dox treated mice in remission. (a) FSC/SSC profile of AML-derived cells harvested from the spleen of representative AML246-Cherry leukemic mice during an 18 day Dox treatment time course (same mice shown in Fig. 3a), showing viable AML-derived cells identified based on surface CD45.2 and mCherry. (b) Summary of IVIS organ imaging of 16-21 day Dox treated mice. (c) Flow cytometry analysis of AML-derived cells identified based on surface CD45.2 and mCherry in the bone marrow, spleen, and liver of mouse #40, showing early relapsing mCherry^{+}GFP^{HIGH}SiglecF$^{–}$ cells specifically in the spleen. Note that

bone marrow and liver are gated on $mCherry^{+}GFP^{-}$cells, whereas spleen is gated on $mCherry^{+}GFP^{+}$ early relapse cells. (d) Flow cytometry analysis of bone marrow, liver, and spleen from day 21 Dox treated mouse #34 with AML-derived cells identified based on surface CD45.2 and mCherry. Note that bone marrow and liver are gated on $mCherry^{+}GFP^{-}$cells, whereas spleen is gated on $mCherry^{+}GFP^{+}$ early relapse cells. (e) IVIS mCherry imaging of organs from day 21 Dox treated mouse #3742 where $GFP^{+}mCherry^{+}$ early relapse cells were detected in the spleen by flow cytometry, showing mCherry signal restricted to the spleen, kidneys and liver. At each time point leukemic mouse organs were imaged alongside matched control organs from a non-transplanted $Rag1^{-/-}$ mouse for signal calibration.

a

b

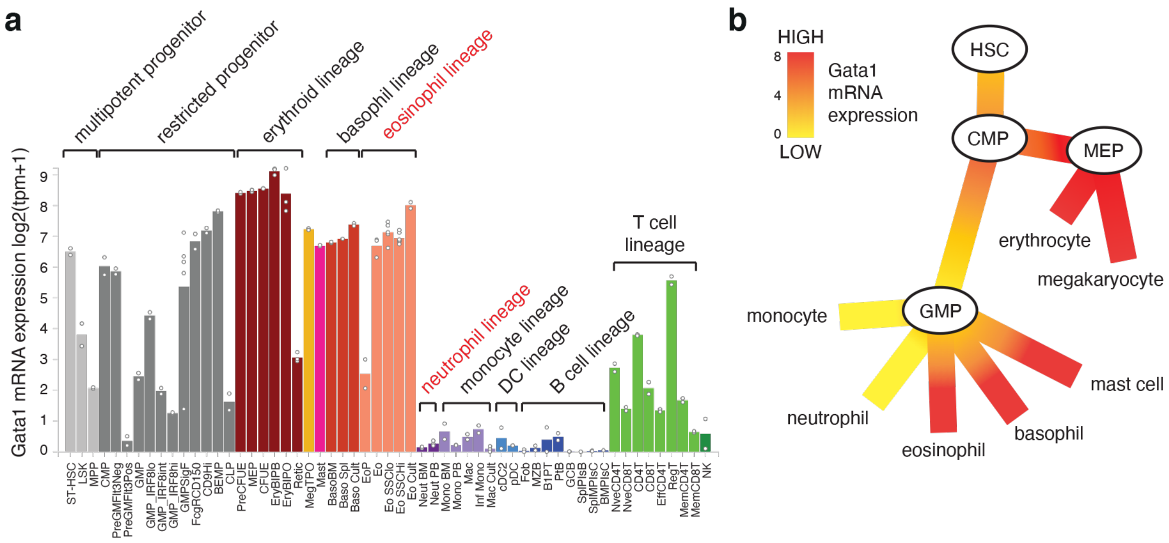

c

gene	clone	alleles		translation	summary
Gata1	sgRNA 2 clone 5	**ATGCTAGCTGGGCCTATGGCAA-GACGGCAC**	**wt**	ASWAYGKTA	wt
		ATGCTAGCTGGGCCTATGGCAAAGACGGCAC	mut1	ASWAYGKDG(12)*	K100FsX14
		ATGCTAGCTGGGCCTATGGCAAAGACGGCAC	mut2	ASWAYGKDG(12)*	K100FsX14
Gata1	sgRNA 3 clone 5	**TTCCCAGGCCCTGGAAGACCAGGAAGGGAA**	**wt**	SQALEDQEG	wt
		TTCCCAGGCCCTGGAAGA-CAGGAAGGGAA	mut1	SQALEDRKG(10)*	D123FsX13
		TTCCCAGG--------------GAAGGGAA	mut2	SQGREEQQH(36)*	Q119FsX43
Gata1	sgRNA 3 clone 8	**TTCCCAGGCCCTGGAAGACCA-GGAAGGGAA**	**wt**	SQALEDQEG	wt
		TTCCCAGGCCCTGGAAGACCAAGGAAGGGAA	mut1	SQALEDQGR(41)*	Q124FsX43
		TTCCCAGGCCCTGGAAGACCAAGGAAGGGAA	mut2	SQALEDQGR(41)*	Q124FsX43

d

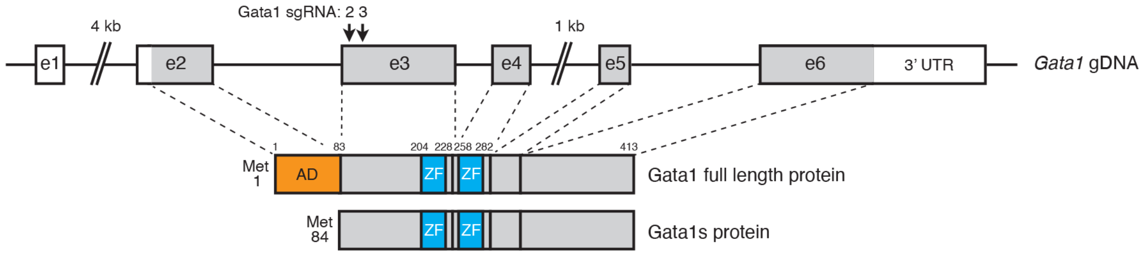

Supplementary Figure 5. Characterization of Gata1 knockout AML246-Cherry clones. (a) Mouse Gata1 expression across hematopoietic cell types from Haemosphere, associated with the Haemopedia database [2]. (b) Schematic representation of mouse Gata1 expression across myeloid lineages based on data from a. (c) Sequence analysis of three independent AML246-Cherry Gata1 KO clones showing biallelic genomic DNA mutations (mut1 and mut2) and their predicted protein products. sgRNA target sites are blue and PAM sequences are green. (d) Schematic of the *Gata1* gene and its encoded full length Gata1 and Gata1s proteins.

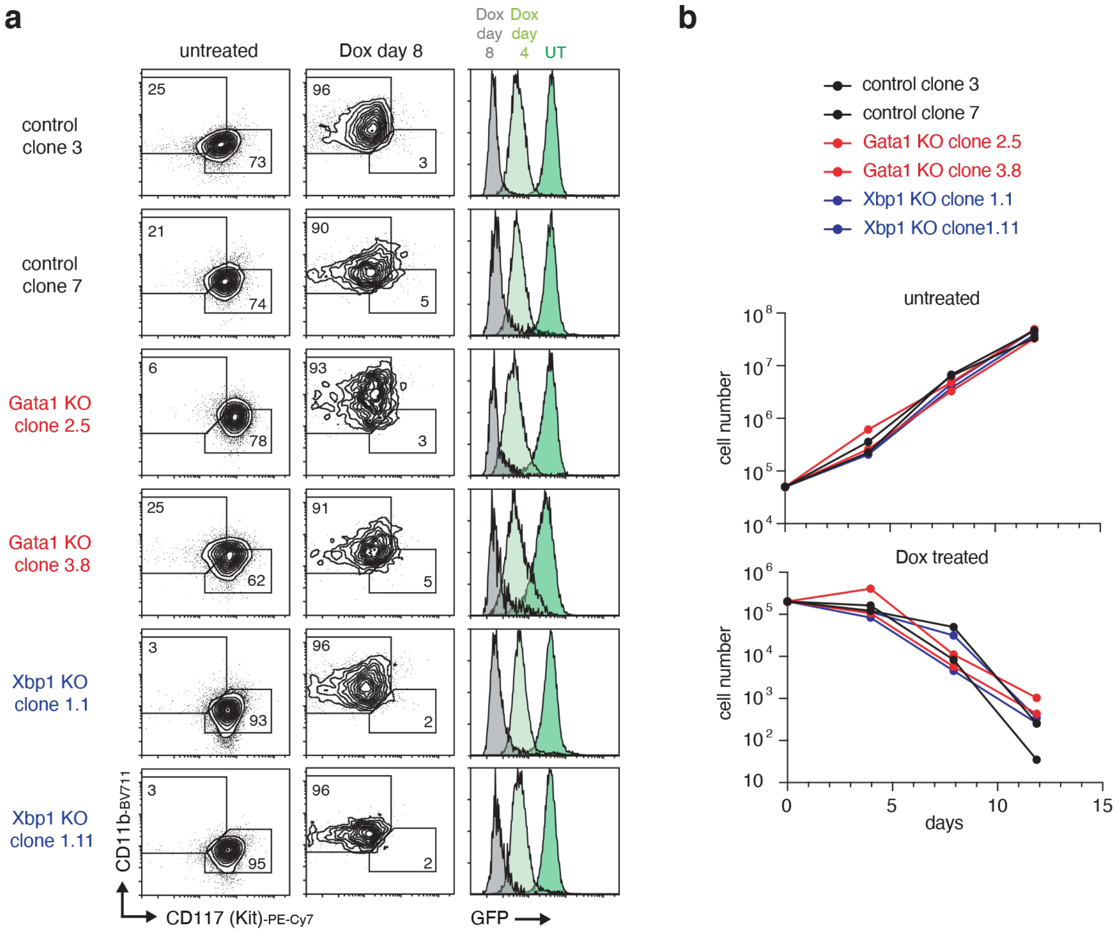

Supplementary Figure 6. Gata1 and Xbp1 knockout clone analysis *in vitro*. (a) Flow cytometry analysis of the Dox response of clones in culture, showing similar CD11b induction, CD117 (Kit) repression, and GFP repression for control, Gata1, and Xbp1 knockout AML246 cells. (b) Time course showing proliferation of untreated and Dox-treated clones in culture. Average of 3 technical replicates per clone.

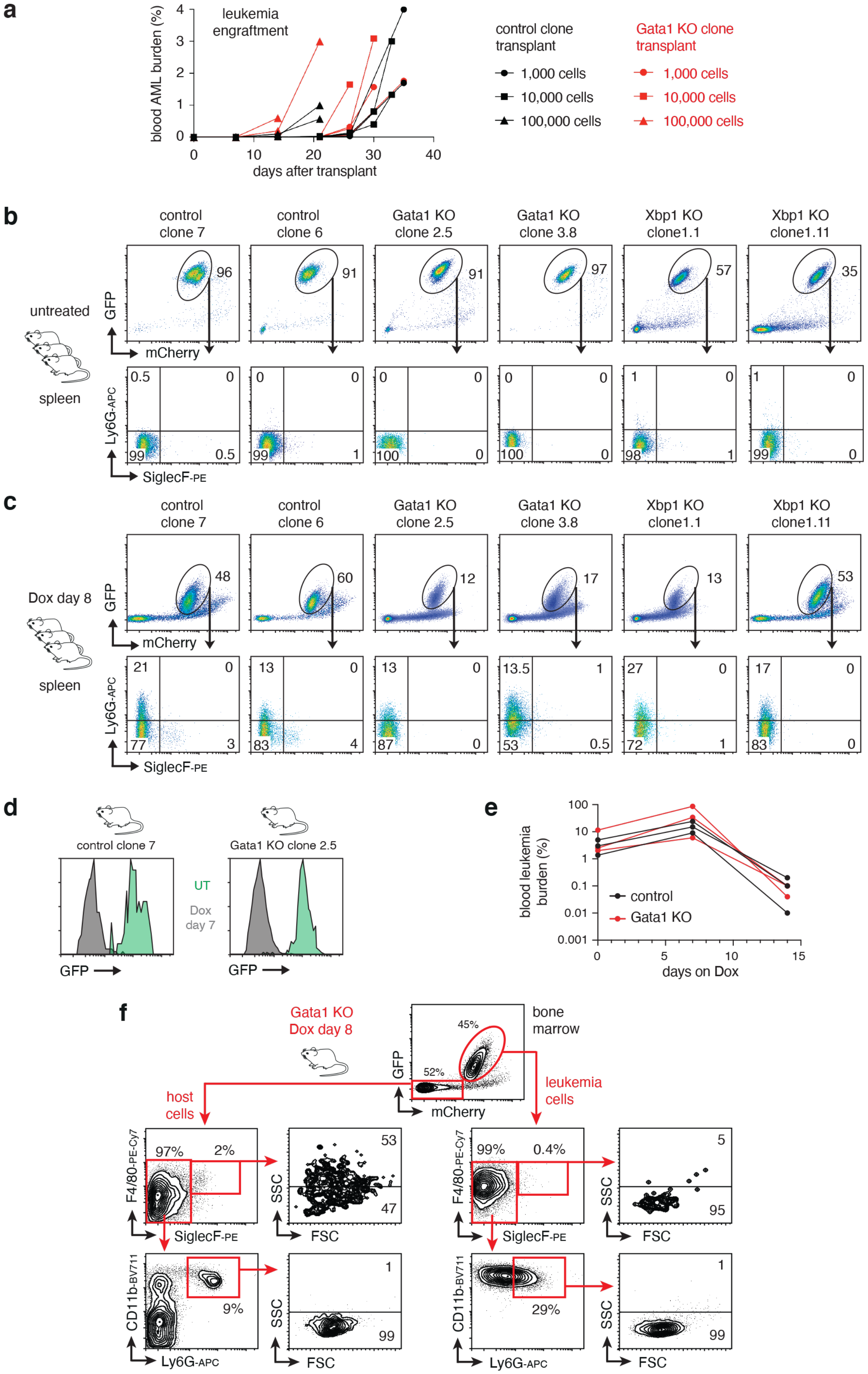

Supplementary Figure 7. Gata1 and Xbp1 knockout clone analysis *in vivo*. (a) Blood leukemia burden indicating engraftment following transplant of a dilution series of control and Gata1 knockout AML246 cells as indicated. Two mice were transplanted for each condition. (b-c) Flow

cytometry analysis of splenocytes from mice with established disease following transplant of the indicated control or knockout clones, then either untreated (b) or following 8 days Dox (c). Leukemia gates are indicated in the upper plots. (d) Peripheral blood GFP profile of mice transplanted with the indicated clones and left untreated (UT; green) or Dox treated for 7 days (grey). (e) Leukemia burden in the blood of mice with established disease following transplant of control or Gata1 knockout clones then treated with Dox over a 14 day time course. (F) Flow cytometry analysis of a representative *Rag1*$^{-/-}$ mouse with established Gata1 KO AML246-Cherry after 8 days Dox treatment, comparing the immunophenotype of AML-derived (mCherry^{+}) populations to host (mCherry^{-}) neutrophils (SSC^{LOW}Ly6G^{+}) and eosinophils (SSC^{HIGH}SiglecF^{+}).

a

gene	clone	alleles		predicted translation	summary
Xbp1	3.1	**ACCAGGAGTTAAGAACACGCTTGGGAATGGACACGCTGG**	**wt**	QELRTRLGMDTLDP	wt
		ACCA---------------------AATGGACACGCTGG	mut 1	QDTLDPDEVPEVEA(227)	Q124delX241
		ACCAGGA-------------------ATGGACACGCTGG	mut 2	QEWTRWILTRFQRW(7)	E125FsX19
Xbp1	3.11	**ACCAGGAGTTAAGAACACGCTTGGGAATGGACACGCTGG**		QELRTRLGMDTLDP	wt
		ACCAGGAGTTAAG-----------GAATGGACACGCTGG	mut 1	QELRNGHAGS-	R127FsX7
		ACCAGGAGTTAAGAACACGC----------------TGG	mut 2	QELRTRWILTRFQR(8)	R129FsX16

b

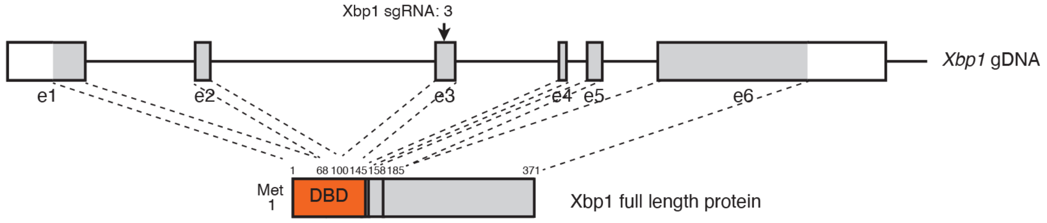

Supplementary Figure 8. Characterization of Xbp1 knockout AML246-Cherry clones. (a) Sequence analysis of two independent AML246-Cherry Xbp1 KO clones showing biallelic (mut1 and mut2) genomic DNA mutations and their predicted protein products. sgRNA target sites are blue and PAM sequences are green. (b) Schematic of the *Xbp1* gene and its encoded protein.

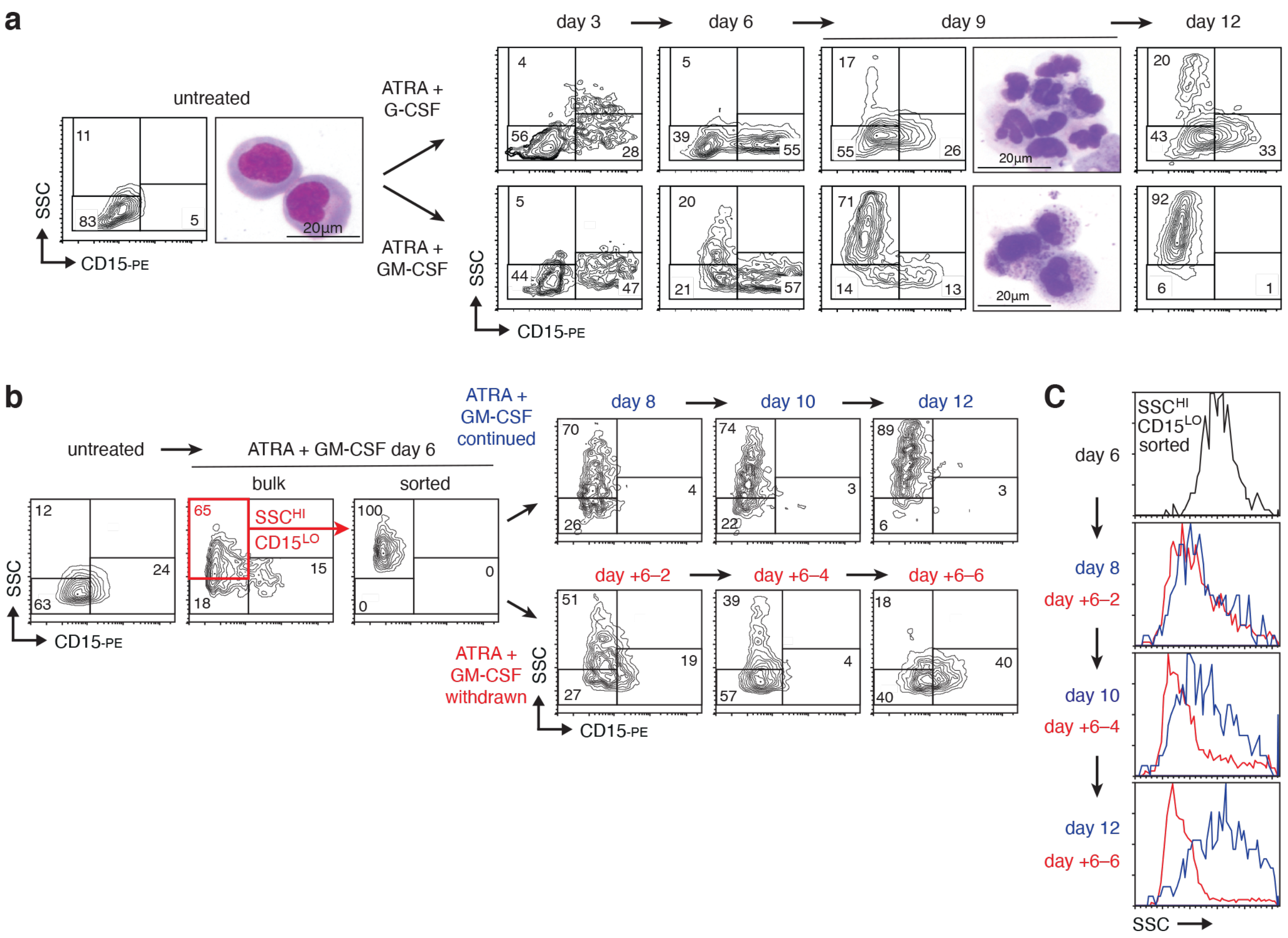

Supplementary Figure 9. Reversion of mature human HT93-derived eosinophils to an immature proliferative state. (a) SSC/CD15 profiles (matching the SSC/CD11B profiles in Fig. 8a) of HT93 cells during ATRA treatment combined with G-CSF (upper panels) or GM-CSF (lower panels). Representative cytospins are shown for untreated (UT) and ATRA day 9 treatments. This experiment was performed once. (b) SSC/CD15 profiles (matching the SSC/CD11B profiles in Fig. 8b) of $SSC^{HIGH}CD11B^{+}CD15^{-}$ cells sorted from ATRA+GM-CSF cultures then either maintained on treatment (upper panels) or following ATRA/GM-CSF withdrawal (lower panels). (c) SSC profile of cells from b, showing progressive return to an immature state following ATRA/GM-CSF withdrawal.

SUPPLEMENTARY REFERENCES

1. McKenzie MD*, et al.* Interconversion between Tumorigenic and Differentiated States in Acute Myeloid Leukemia. *Cell Stem Cell* **25**, 258-272 e259 (2019).

2. Choi J*, et al.* Haemopedia RNA-seq: a database of gene expression during haematopoiesis in mice and humans. *Nucleic Acids Res* **47**, D780-D785 (2019).

Supplementary Table 1. Mice transplanted with AML246-Cherry used in this study.

mouse ID	Figures	time point
3393	1B	Dox day -14
77	1B	UT
3391	1B, S1B	Dox day 7
3410	1B	Dox day 14
3316	1B	Dox day 35
3315	1B-1D, S1B	relapse
3341	1C, 1D,S1A, S1B	relapse
3158	1C, 1D, S1B	relapse
3589	1C	relapse
3327	1C, 4A	relapse
3302	1C	relapse
3324	1C	relapse
3568	1C	relapse
3592	1C	relapse
3591	1C	relapse
3440	1C	relapse
8 (sn25)	1C	relapse
3584	1C	relapse
3687	2B, S2B, S2C, S3A, S3B	Dox day 8
4443	2C, S2A	Dox day 10
82	2A	UT
31	2D	UT
12	2D	UT
20	2D	Dox day 5
23	2D	Dox day 5
7	3D, 4A	Dox day 10
3920	3A-3D, 4A	UT
1	3B	UT
3918	3B, S4A	UT
3692	3A-3B, S3B,S4A	Dox day 8
3693	3B, 4A, S3B	Dox day 8
3762	3B,S4B	Dox day 16
3763	3A,3B,4B, S4A,S4B	Dox day 16
3768	S3B	Dox day 8
30	S3B	Dox day 8
3784	S3B	Dox day 8
3	S3B	Dox day 8
27	S3B	Dox day 8
0	S3B	Dox day 8
4151	S3C	UT
2	S3C	Dox day 10
4	S3D	Dox day 5
4588	4A, 4B, S4B	Dox day 16
4589	4B,S4B	Dox day 16
39	4B,4C	Dox day 16
40	4B,4C,S4C	Dox day 16
3749	3A,3B,4B,4C,S4A, S4B	Dox day 18
3761	3B,3D,4A,4B S4B	Dox day 18
3742	4B,S4B	Dox day 21
3748	4B,S4B	Dox day 21
1 (sn23)	4B	Dox day 21
2 (sn23)	4B	Dox day 21
5 (sn23)	4B	Dox day 21
45	4A,4B	Dox day 21
34	4B,S4D	Dox day 21
41	4B	Dox day 21
3404	S1A, S1B	UT
3501	S1B	UT
3298	S1B	UT
3403	S1A, S1B	Dox day 7
3406	S1B	Dox day 7
4446	S1C	UT
4444	S1C	Dox day 7
1 (sn25)	S1C, S1E	UT and KM
2 (sn25)	S1C, S1E	UT and KM
3 (sn25)	S1C, S1E	UT and KM
4 (sn25)	S1C, S1D	UT and Dox day 7
5 (sn25)	S1C,S1D	UT and KM
6 (sn25)	S1C	UT
7 (sn25)	S1C, S1D	UT and Dox day 7
8 (sn25)	S1C, S1E	UT and KM
9 (sn25)	S1C, S1D	UT and Dox day 7
10 (sn25)	S1C, S1E	UT and KM
11 (sn25)	S1C, S1D	UT and Dox day 7
12 (sn25)	S1C, S1D	UT and Dox day 7
13 (sn25)	S1C, S1E	UT and KM
14 (sn25)	S1C, S1E	UT and KM
15 (sn25)	S1C, S1E	UT and KM
4446	S2A	UT
4444	S2A	Dox day 7

Supplementary Table 2. Detection of early relapse mCherry+GFP+ cells following 7-14 day Dox treatment. The number of GFP+ cells per total cells assessed by flow cytometry within each organ is shown. The GFP+ threshold was set using a GFP-HIGH control sample harvested from an untreated mouse on the same day, adjusted so that 96% of these cells were in the GFP+ gate. Dox-treated mice where mCherry+GFP+ cells were part of a GFP continuum at the tail end of the bulk leukemia profile (prevalent at Dox day 7 where bulk GFP repression is incomplete) in at least one of the tissues analyzed are indicated in blue. Dox-treated mice where mCherry+GFP+ cells were a discrete population clearly distinct from the bulk leukemia (prevalent at Dox days 10-14 where the bulk leukemia is GFP-LOW) in at least one of the tissues analyzed are indicated in red.

Dox day 7	number of mCherry+GFP+ cells		
mouse ID	blood	bone marrow	spleen
3406	0/10,000	0/11,000	0/13,000
3391	0/9000	1/11,000	0/14,000
3903	ND	1/10,000	1/12,000
3693	0/40,000	0/60,000	0/210,000
3692	0/21,000	1/51,000	1/215,000
3687	0/26,000	0/55,000	0/175,000
10	1/11,000	0/10,000	0/10,000
3907	0/10,000	15/230,000	27/30,000
3900	0/10,000	19/130,000	35/200,000
3899	0/11,000	34/220,000	18/64,000
3932	ND	0/90,000	0/13,000
3924	ND	1/20,000	1/16,000
4444	0/200,000	30/1,000,000	14/740,000
**Dox day 10**	**number of mCherry+GFP+ cells**		
**mouse ID**	**blood**	**bone marrow**	**spleen**
20	0/76,000	2/22,000	2/200,000
3793	0/150,000	0/370,000	0/150,000
24	ND	1/470,000	0/72000
5	0/15,000	0/350,000	0/350,000
6	0/25,000	0/276,000	1/161,000
4	0/55,000	2/755,000	1/150,000
13	0/8,000	1/8,000	0/13,000
18	0/8,000	5/9,000	0/8,000
3503	0/64,000	0/60,000	0/20,000
3390	0/5,000	0/20,000	0/40,000
3380	0/8,000	0/11,000	0/21,000
3405	0/5,000	0/21,000	0/26,000
4569	ND	5/719,000	0/132,000
4443	0/130,000	35/2,000,000	11/600,000
4603	0/300,000	5/160,000	10/3,750,000
**Dox day 14**	**number of mCherry+GFP+ cells**		
**mouse ID**	**blood**	**bone marrow**	**spleen**
3410	0/9,000	0/11,000	0/17,000
3289	0/11,000	0/5,000	1/13,000
3395	0/55,000	0/11,000	1/20,000
3764	1/400,000	239/1,000,000	60/300,000
3757	0/340,000	20/1,000,000	30/500,000
4567	2/23,000	6/1,000,000	130/300,000
4568	0/8,000	3/1,000,000	170/800,000

Supplementary Table 3. Mice transplanted with AML246-Cherry control sgRNA leukemia, Gata1 or Xbp1 KO leukemia in this study.

control				Gata1 KO				Xbp1 KO			
mouse ID	clone	Figures	time point	mouse ID	clone	Figures	time point	mouse ID	clone	Figures	time point
7	sgCON cl6	6B-6D, S7C	Dox day 8	19	Gata1 sgRNA 3 cl8	6C-6D	Dox day 8	18	Xbp1 sgRNA 1 cl 11	S7B	UT
13	sgCON cl7	6C-6D	Dox day 8	20	Gata1 sgRNA 2 cl5	6C-6D, S7C	Dox day 8	11	Xbp1 sgRNA 1 cl 1	S7B	UT
9	sgCON cl6	6C-6D	Dox day 8	21	Gata1 sgRNA 3 cl8	6B-6D,S7C, S7F	Dox day 8	14	Xbp1 sgRNA 1 cl 11	6B-6D S7C	Dox Day 8
13	sgCON cl3	6C-6D	Dox day 8	22	Gata1 sgRNA 2 cl5	6B-6D	Dox day 8	16	Xbp1 sgRNA 1 cl 11	6C-6D	Dox Day 8
1	sgCON cl3	6C-6D, S7C	Dox day 8	4377	Gata1 sgRNA 3 cl6	6C-6D	Dox day 8	8	Xbp1 sgRNA 1 cl 1	6C-6D, S7C	Dox Day 8
12	sgCON cl6	S7B	UT	23	Gata1 sgRNA 3 cl8	S7B	UT	17	Xbp1 sgRNA 1 cl 1	6C-6D	Dox Day 8
1 (sn92)	sgCON cl7	S7A,S7D-S7E	UT and Dox day 8	24	Gata1 sgRNA 2 cl5	S7B	UT	43	Xbp1 sgRNA 1 cl 1	7B-7C	KM
2 (sn92)	sgCON cl7	S7A	UT	7	Gata1 sgRNA 2 cl5	S7A, S7D-S7E	UT and Dox day 8	44	Xbp1 sgRNA 1 cl 1	7B-7C	KM
3 (sn92)	sgCON cl7	S7A,S7E	UT and Dox day 8	11	Gata1 sgRNA 2 cl5	A	UT and Dox day 8	45	Xbp1 sgRNA 1 cl 1	7B-7C	KM
4 (sn92)	sgCON cl7	S7A,S7E	UT and Dox day 8	12	Gata1 sgRNA 2 cl5	S7A, S7D	UT and Dox day 8	46	Xbp1 sgRNA 1 cl 1	7B-7C	KM
5 (sn92)	sgCON cl7	S7A	UT	8	Gata1 sgRNA 2 cl5	S7A	UT	47	Xbp1 sgRNA 1 cl 1	7B-7C	KM
6 (sn92)	sgCON cl7	S7A	UT	9	Gata1 sgRNA 2 cl5	S7A	UT	48	Xbp1 sgRNA 1 cl 1	7B-7C	KM
3	sgCON cl6	7A-7C	KM	10	Gata1 sgRNA 2 cl5	S7A	UT	4454	Xbp1 sgRNA 1 cl 11	7B-7C	KM
4	sgCON cl6	7A-7C	KM	32	Gata1 sgRNA 2 cl5	7A-7C	KM	3	Xbp1 sgRNA 1 cl 11	7B-7C	KM
28	sgCON cl6	7A-7C	KM	33	Gata1 sgRNA 2 cl5	7A-7C	KM	4	Xbp1 sgRNA 1 cl 11	7B-7C	KM
29	sgCON cl6	7A-7C	KM	25	Gata1 sgRNA 2 cl5	7A-7C	KM	5	Xbp1 sgRNA 1 cl 11	7B-7C	KM
30	sgCON cl6	7A-7C	KM	30	Gata1 sgRNA 2 cl5	7A-7C	KM	6	Xbp1 sgRNA 1 cl 11	7B-7C	KM
25	sgCON cl7	7A-7C	KM	28	Gata1 sgRNA 2 cl5	7A-7C	KM	4457	Xbp1 sgRNA 1 cl 11	7B-7C	KM
20	sgCON cl7	7A-7C	KM	26	Gata1 sgRNA 2 cl5	7A-7C	KM				
16	sgCON cl3	7A-7C	KM	27	Gata1 sgRNA 2 cl5	7A-7C	KM				
4396	sgCON cl3	7A-7C	KM	31	Gata1 sgRNA 2 cl5	7A-7C	KM				
11	sgCON cl3	7A-7C	KM	6	Gata1 sgRNA 3 cl8	7A-7C	KM				
14	sgCON cl3	7A-7C	KM	15	Gata1 sgRNA 3 cl8	7A-7C	KM				
15	sgCON cl3	7A-7C	KM	16	Gata1 sgRNA 3 cl8	7A-7C	KM				
4379	sgCON cl3	7A-7C	KM	17	Gata1 sgRNA 3 cl8	7A-7C	KM				
2	sgCON cl6	7A-7C	KM	18	Gata1 sgRNA 3 cl8	7A-7C	KM				
23	sgCON cl7	7A-7C	KM	19	Gata1 sgRNA 3 cl8	7A-7C	KM				
27	sgCON cl7	7A-7C	KM	4424	Gata1 sgRNA 3 cl6	7A-7C	KM				
21	sgCON cl7	7A-7C	KM	4425	Gata1 sgRNA 3 cl6	7A-7C	KM				
22	sgCON cl7	7A-7C	KM	4426	Gata1 sgRNA 3 cl6	7A-7C	KM				
1	sgCON cl6	7A-7C	KM	4428	Gata1 sgRNA 3 cl6	7A-7C	KM				
4466	sgCON cl7	7A-7C	KM	4394	Gata1 sgRNA 3 cl6	7A-7C	KM				
4458	sgCON cl3	7A-7C	KM								

**Supplementary Table 4. Primer and sgRNA sequences.** All sequences are 5' to 3'.	
Description	Sequence
Control sgRNA	Forward: caccTAGCGAACGTGTCCGGCG<br>Reverse: aaacCGCCGGACACGTTCGCTA
Gata1 sgRNA 2	Forward: caccGCTGGGCCTATGGCAAGA<br>Reverse: aaacTCTTGCCATAGGCCCAGC
Gata1 sgRNA 3	Forward: caccGGCCCTGGAAGACCAGGA<br>Reverse: aaacTCCTGGTCTTCCAGGGCC
Gata1 gDNA PCR	Forward: AACTCCGAAGTCACCCAAGCAG<br>Reverse: AGGATGGGAGAACATGGGTT
Gata1 cDNA PCR	Forward: GTTCAACCCCAGTGTTCCCA<br>Reverse: AAGGGGCCAATGGCAGG
Gata1 RT-qPCR	Forward: GGGATCACCCTGAACTCGTC<br>Reverse: GGTTGAACCTGGGCTTGTTG
Rn18s RT-qPCR	Forward: GTAACCCGTTGAACCCCATT<br>Reverse: CCATCCAATCGGTAGTAGCG
SiglecF RT-qPCR	Forward: GGTCTCACAGGTGAAGGTCC<br>Reverse: GGCAAGATGGTTGCCTTTCG
Prg3 RT-qPCR	Forward: CCCTTGGGTAGTGAGTGCTG<br>Reverse: GTCCAACGCAGCTTCTATGC
Epx RT-qPCR	Forward: CTGCTTAGCTGTAGTGGGGG<br>Reverse: TGTGAGCACATCAGTGGCAT
Ly6G RT-qPCR	Forward: AGAGGAAGTTTTATCTGTGCAGCC<br>Reverse:TCAGGTGGGACCCCAATACA
Coro1a RT-qPCR	Forward: GGGCTGAGTCCCCCATTAAG<br>Reverse: GAGACGCGCACATCCTCATA
Spi1 RT-qPCR	Forward: CTGGAGCTCAGCTGGATGTTAC<br>Reverse: GCCATCAGCTTCTCCATCAGA
Xbp1 sgRNA 3	Forward: caccGGAGTTAAGAACACGCTT<br>Reverse: aaacAAGCGTGTTCTTAACTCC
Xbp1 gDNA PCR	Forward: CTCTGTCCCATTAGCCACCG<br>Reverse:TGAATTTTCCCTGTTTCCTTGAACT

**Supplementary Table 5. shRNA oligonucleotide sequences.** All sequences are 5' to 3'.	
shRNA	Oligonucleotide sequence
Renilla.713	Forward:<br>TCGAGAAGGTATATTGCTGTTGACAGTGAGCGCAGGAATTATAATGCTTATCTATAGTGAAGC<br>CACAGATGTATAGATAAGCATTATAATTCCTATGCCTACTGCCTCGGACTTCAAGGGGCTAG
	Reverse:<br>AATTCTAGCCCCTTGAAGTCCGAGGCAGTAGGCATAGGAATTATAATGCTTATCTATACATCTGT<br>GGCTTCACTATAGATAAGCATTATAATTCCTGCGCTCACTGTCAACAGCAATATACCTTC
Gata1.1377	Forward:<br>TCGAGAAGGTATATTGCTGTTGACAGTGAGCGCCTGTAGCTATGTAGCTATGAATAGTGAAGC<br>CACAGATGTATTCATAGCTACATAGCTACAGATGCCTACTGCCTCGGACTTCAAGGGGCTAG
	Reverse:<br>AATTCTAGCCCCTTGAAGTCCGAGGCAGTAGGCATCTGTAGCTATGTAGCTATGAATACATCTG<br>TGGCTTCACTATTCATAGCTACATAGCTACAGGCGCTCACTGTCAACAGCAATATACCTTC
Gata1.1388	Forward:<br>TCGAGAAGGTATATTGCTGTTGACAGTGAGCGATAGCTATGAACTATGTAGATATAGTGAAGC<br>CACAGATGTATATCTACATAGTTCATAGCTACTGCCTACTGCCTCGGACTTCAAGGGGCTAG
	Reverse:<br>AATTCTAGCCCCTTGAAGTCCGAGGCAGTAGGCAGTAGCTATGAACTATGTAGATATACATCTG<br>TGGCTTCACTATATCTACATAGTTCATAGCTATCGCTCACTGTCAACAGCAATATACCTTC
