# Supplementary material for: Acute myeloid leukemia maturation lineage influences residual disease and relapse following differentiation therapy
Source: Nat Commun. 2021 Nov 11;12:6546. doi: 10.1038/s41467-021-26849-w (PMC8586014; doi:10.1038/s41467-021-26849-w)
Supplement: Supplementary file 2 — Reporting summary [file 41467_2021_26849_MOESM2_ESM.pdf]

nature portfolio

Corresponding author(s): Ross Dickins
Last updated by author(s): Oct 4, 2021

Reporting Summary

Nature Portfolio wishes to improve the reproducibility of the work that we publish. This form provides structure for consistency and transparency in reporting. For further information on Nature Portfolio policies, see our Editorial Policies and the Editorial Policy Checklist.

Statistics

For all statistical analyses, confirm that the following items are present in the figure legend, table legend, main text, or Methods section.

n/a	Confirmed	
☐	☒	The exact sample size (*n*) for each experimental group/condition, given as a discrete number and unit of measurement
☐	☒	A statement on whether measurements were taken from distinct samples or whether the same sample was measured repeatedly
☐	☒	The statistical test(s) used AND whether they are one- or two-sided<br>*Only common tests should be described solely by name; describe more complex techniques in the Methods section.*
☒	☐	A description of all covariates tested
☐	☒	A description of any assumptions or corrections, such as tests of normality and adjustment for multiple comparisons
☐	☒	A full description of the statistical parameters including central tendency (e.g. means) or other basic estimates (e.g. regression coefficient) AND variation (e.g. standard deviation) or associated estimates of uncertainty (e.g. confidence intervals)
☐	☒	For null hypothesis testing, the test statistic (e.g. *F*, *t*, *r*) with confidence intervals, effect sizes, degrees of freedom and *P* value noted<br>*Give P values as exact values whenever suitable.*
☒	☐	For Bayesian analysis, information on the choice of priors and Markov chain Monte Carlo settings
☒	☐	For hierarchical and complex designs, identification of the appropriate level for tests and full reporting of outcomes
☒	☐	Estimates of effect sizes (e.g. Cohen's *d*, Pearson's *r*), indicating how they were calculated

Our web collection on statistics for biologists contains articles on many of the points above.

Software and code

Policy information about availability of computer code

Data collection	Flow cytometry data was collected on either on BD LSRII or LSRFortessa (BD Biosciences), using DiVA V6.3.1 .<br>RT-PCR reactions were performed using a LightCycler 480 (Roche).<br>Western Blot imaging was performed on a ChemiDoc Touch Imaging System (BioRad)<br>IVIS imaging data was collected using the IVIS Lumina Living Image software 4.5.2.<br>Aperio ImageScope (Leica).
Data analysis	GraphPad Prism 8 (GraphPad Software, USA).<br>Flowlogic software 7.2.1 (Inivai Technologies, Australia).

For manuscripts utilizing custom algorithms or software that are central to the research but not yet described in published literature, software must be made available to editors and reviewers. We strongly encourage code deposition in a community repository (e.g. GitHub). See the Nature Portfolio guidelines for submitting code & software for further information.

Data

Policy information about availability of data

All manuscripts must include a data availability statement. This statement should provide the following information, where applicable:

- Accession codes, unique identifiers, or web links for publicly available datasets
- A description of any restrictions on data availability
- For clinical datasets or third party data, please ensure that the statement adheres to our policy

The authors declare that the data supporting the findings of this study are available within the paper and its supplementary information files. All unique materials are readily available from the authors.

Field-specific reporting

Please select the one below that is the best fit for your research. If you are not sure, read the appropriate sections before making your selection.

☒ Life sciences ☐ Behavioural & social sciences ☐ Ecological, evolutionary & environmental sciences

For a reference copy of the document with all sections, see nature.com/documents/nr-reporting-summary-flat.pdf

Life sciences study design

All studies must disclose on these points even when the disclosure is negative.

Sample size	We followed conventions in the field and our previous experience to determine sample sizes. No power analysis was carried out. Mouse cohort sizes for each experiment were based on our previous studies (see McKenzie et al 2019 Cell Stem Cell doi 10.1016/j.stem.2019.07.001; Witkowski et al 2017 J Exp Med doi 10.1084/jem.20160048; Liu et al 2014 Genes Dev doi 10.1101/gad.240416.114).
Data exclusions	Data from certain mice were excluded due to unexpected/unexplained deaths but these were sporadic and stochastic.
Replication	Replicates are indicated in the figure legends and/or methods. Most assays were performed in triplicate.
Randomization	Randomization was not relevant to our experimental design because all animals were transplanted equivalently and Dox treatments were usually initiated upon mice reaching a certain disease burden rather than on a group-by-group basis.
Blinding	Blinding was not incorporated into our experimental design because for in vivo studies mice receiving leukemia transplants must be clearly documented to allow careful animal ethics monitoring. Researchers were aware which mice had been transplanted and Dox treated, because Dox chow was provided on a cage-by-cage basis incompatible with blinding.

Reporting for specific materials, systems and methods

We require information from authors about some types of materials, experimental systems and methods used in many studies. Here, indicate whether each material, system or method listed is relevant to your study. If you are not sure if a list item applies to your research, read the appropriate section before selecting a response.

Materials & experimental systems

n/a	Involved in the study	
☐	☒	Antibodies
☐	☒	Eukaryotic cell lines
☒	☐	Palaeontology and archaeology
☐	☒	Animals and other organisms
☒	☐	Human research participants
☒	☐	Clinical data
☒	☐	Dual use research of concern

Methods

n/a	Involved in the study	
☒	☐	ChIP-seq
☐	☒	Flow cytometry
☒	☐	MRI-based neuroimaging

Antibodies

Antibodies used	MOUSE antigens were detected using the following antibodies:<br>CD11b-BV711 (clone M1/70, catalog no. 563168, eBioscience)<br>Ly6G-APC (clone 1A8-Ly6G, catalog no. 127614, eBioscience)<br>F4/80-PE/Cy7 (clone BM8, catalog no. 123113 eBioscience)<br>SiglecF-PE (clone E50-2440, catalog no. 562068, BD Biosciences)<br>Ccr3-BV421 (clone J073E5, catalog no. 144517, Biolegend)

	Thy1.1-APC (clone OX-7, catalog no. 202526 ,BioLegend)<br>CD45.1-AF700 (clone A20, catalog no. 110723, BD Biosciences)<br>CD45.2-V450 (clone 104, catalog no. 560697, BD Biosciences)<br>Alpha-tubulin (clone DM1A catalog no. T9026, Sigma Aldrich).<br><br>HUMAN antigens were detected using the following antibodies:<br>CD15-PE (clone W6D3, catalog no. 323005, Biolegend)<br>CD11b-APC (clone Bear1, catalog no. A87782, Beckman Coulter)
Validation	Flow cytometry antibodies in this study are well characterized, with validation data provided by the commercial supplier as linked below:<br><br>MOUSE:<br>CD11b-BV711 (clone M1/70, catalog no. 563168, eBioscience). www.bdbiosciences.com/en-eu/products/reagents/flow-cytometry-reagents/research-reagents/single-color-antibodies-ruo/bv711-rat-anti-cd11b.563168.<br>Ly6G-APC (clone 1A8-Ly6G, catalog no. 127614, eBioscience). www.biolegend.com/en-us/products/apc-anti-mouse-ly-6g-antibody-6115?GroupID=BLG7234.<br>F4/80-PE/Cy7 (clone BM8, catalog no. 123113 eBioscience). www.biolegend.com/en-us/products/pe-cyanine7-anti-mouse-f4-80-antibody-4070?GroupID=BLG5319.<br>SiglecF-PE (clone E50-2440, catalog no. 562068, BD Biosciences). www.bdbiosciences.com/en-us/products/reagents/flow-cytometry-reagents/research-reagents/single-color-antibodies-ruo/pe-rat-anti-mouse-siglec-f.562068.<br>Ccr3-BV421 (clone J073E5, catalog no. 144517, Biolegend). www.biolegend.com/en-us/products/brilliant-violet-421-anti-mouse-cd193-ccr3-antibody-11896?GroupID=BLG11057.<br>Thy1.1-APC (clone OX-7, catalog no. 202526 ,BioLegend). www.biolegend.com/en-us/products/apc-anti-rat-cd90-mouse-cd90-1-thy-1-1-antibody-5621?GroupID=BLG10566.<br>CD45.1-AF700 (clone A20, catalog no. 110723, BD Biosciences). www.biolegend.com/en-us/products/alexa-fluor-700-anti-mouse-cd45-1-antibody-3392?GroupID=BLG1933. https://www.bdbiosciences.com/en-eu/products/reagents/flow-cytometry-reagents/research-reagents/single-color-antibodies-ruo/v450-mouse-anti-mouse-cd45-2.560697.<br>CD45.2-V450 (clone 104, catalog no. 560697, BD Biosciences). www.bdbiosciences.com/en-eu/products/reagents/flow-cytometry-reagents/research-reagents/single-color-antibodies-ruo/v450-mouse-anti-mouse-cd45-2.560697.<br>Alpha-tubulin (clone DM1A catalog no. T9026, Sigma Aldrich). www.sigmaaldrich.com/AU/en/product/sigma/t9026?context=product.<br><br>HUMAN:<br>CD15-PE (clone W6D3, catalog no. 323005, Biolegend). www.biolegend.com/en-us/products/pe-anti-human-cd15-ssea-1-antibody-3701?GroupID=BLG4807.<br>CD11b-APC (clone Bear1, catalog no. A87782, Beckman Coulter). www.beckman.com/reagents/coulter-flow-cytometry/antibodies-and-kits/single-color-antibodies/cd11b/a87782.

Eukaryotic cell lines

Policy information about cell lines

Cell line source(s)	AML246 cells were generated in-house as described in reference 33. The HT93 cell line was obtained from from the Cell Resource Center for Biomedical Research, Tohoku University, Japan.
Authentication	AML246 cells were not authenticated because they were generated in-house, but Dox-induced differentiation was regularly verified. HT93 cells were authenticated by STR profiling at CellBank Australia.
Mycoplasma contamination	All cell lines regularly tested negative for mycoplasma using a PCR-based protocol.
Commonly misidentified lines<br>(See ICLAC register)	No commonly misidentified cell lines were used in the study.

Animals and other organisms

Policy information about studies involving animals; ARRIVE guidelines recommended for reporting animal research

Laboratory animals	Rag or Ly5.1/Rag mice were used for these studies. All mice were females and ranged from 4-6 weeks of age at commencement of in vivo experiments. Mice were housed with a 12 hour light/dark cycle and standard air conditioned ambient temperature and humidity.
Wild animals	The study did not involve the use of wild animals.
Field-collected samples	The study did not involve the use of samples collected from the field.
Ethics oversight	Animal ethics was approved by Alfred Research Alliance Animal Ethics Committee.

Note that full information on the approval of the study protocol must also be provided in the manuscript.

Flow Cytometry

Plots

Confirm that:

- [x] The axis labels state the marker and fluorochrome used (e.g. CD4-FITC).
- [x] The axis scales are clearly visible. Include numbers along axes only for bottom left plot of group (a 'group' is an analysis of identical markers).
- [ ] All plots are contour plots with outliers or pseudocolor plots.
- [x] A numerical value for number of cells or percentage (with statistics) is provided.

Methodology

Sample preparation	Blood was collected by mandible bleed or tail prick. For analysis of leukemia in mice, single cell suspensions derived from bone marrow, spleen, peripheral blood, and liver were treated with red cell lysis buffer (15 mM NH_4Cl, 1 mM $KHCO_3$, 0.01 mM EDTA) and washed twice in FACS staining buffer (PBS supplemented with 5% FCS) before incubation with fluorochrome-conjugated antibodies. Bone marrow, spleen and liver cells were processed through a 40 um filter to generate single cell suspensions. No tissue processing steps were used for experiments cultured in vitro.
Instrument	Flow cytometry was performed using a BD LSRII or LSRFortessa (BD Biosciences), and cell sorting was performed using a BD Influx (BD Biosciences) with a 100 μm nozzle.
Software	Analysis was performed using Flowlogic software (Inivai Technologies, Australia).
Cell population abundance	50,000-100,000 cells were sorted for the Day 8 Dox cytospins of AML-derived SSChigh and SSClow cells respectively.<br>200,000 SSChigh cells were sorted for the HT93 reversion assay. Purity of the sort was determined by analysing 10% of the sorted populations on the LSRII immediately post-sort.
Gating strategy	Unless otherwise stated in the figure legend, all experiments relating to AML246 are initially gated on FSC/SSC, followed by FSC-A/FSC-H and SSC-H/SSC-A gates to eliminate doublets, followed by a gate drawn on viable cells. For mice harvested at UT, day 5, day 8, day 10 dox and at relapse, AML cells were gated on based on mCherry/GFP expression. For mice harvested at days 16,18 and 21, a cd45.1/cd45.2 pre-gate was drawn prior to mCherry/GFP in order to purify the AML-derived population. CD11b/Ly6g expression and Siglecf/F480/Ccr3 expression was then analysed on the AML-derived population to determine the immunophenotype of the cells.<br>For HT93 related experiments, HT93 cells are initially gated on FSC/SSC, followed by FSC-A/FSC-H and SSC-H/SSC-A gates to eliminate doublets, followed by a gate drawn on viable cells.

- [x] Tick this box to confirm that a figure exemplifying the gating strategy is provided in the Supplementary Information.
